# Transmission, Spread, Longevity and Management of Hop Latent Viroid, a Widespread and Destructive Pathogen Affecting Cannabis (*Cannabis sativa* L.) Plants in North America

**DOI:** 10.3390/plants14050830

**Published:** 2025-03-06

**Authors:** Zamir K. Punja, Cameron Scott, Heather H. Tso, Jack Munz, Liam Buirs

**Affiliations:** 1 Department of Biological Sciences, Simon Fraser University, 8888 University Drive, Burnaby, BC V5A 1S6, Canada; cameron_scott_2@sfu.ca (C.S.); heather_tso@sfu.ca (H.H.T.); lbuirs@puresunfarms.com (L.B.); 2 3 Rivers Biotech, Coquitlam, BC V3K 3A3, Canada; jack.m@3riversbiotech.com; 3 Pure Sunfarms, Delta, BC V4K 3N3, Canada

**Keywords:** *Cannabis sativa*, cannabinoids, dudding, marijuana, stunt disease, hop latent viroid

## Abstract

Hop latent viroid (HLVd), a 256-nucleotide RNA strand with complementary base-pairing and internal stem loop structures, forms circular or rod-shaped molecules within diseased plants. RT-PCR/RT-qPCR was used to assess HLVd transmission, spread and longevity. The viroid was detected in asymptomatic stock plants and in rooted vegetative cuttings, as well as in recirculated nutrient solution sampled from propagation tables and nozzles. Plant-to-plant spread through root infection in hydroponic cultivation was demonstrated. The viroid survived for 7 days and 4 weeks, respectively, in crushed leaf extracts (sap) or dried leaves/roots at room temperature. Following stem inoculation with infectious sap, HLVd was detected in root tissues within 2–3 weeks and in the foliage within 4–6 weeks. Plants grown under a 12:12 h photoperiod to induce inflorescence development showed more rapid spread of HLVd compared to 24 h lighting. The viroid was subsequently detected in inflorescence tissues, in trichome glands, in dried cannabis flowers and in crude resinous oil extracts. Anthers and pollen from infected male plants and seeds from infected female plants contained HLVd, giving rise to up to 100% infected seedlings. Artificially inoculated tomato and tobacco plants supported viroid replication in roots and leaves. Infected cannabis leaf and root tissues treated with UV-C for 3–5 min or temperatures of 70–90 °C for 30 min contained amplifiable HLVd-RNA. Infectious plant extract treated with 5–10% bleach (0.825% NaOCl) or 1000 ppm hypochlorous acid yielded no RT-PCR bands, suggesting the RNA was degraded. Meristem tip culture from HLVd-infected plants yielded a high frequency of pathogen-free plants, depending on the genotype.

## 1. Introduction

The commercial cultivation of high Δ9-tetrahydrocannabinol (THC)-containing cannabis (*Cannabis sativa* L.) plants in Canada and in many US states where it is legally permitted by regulatory jurisdictions is constantly faced with challenges from plant pathogens [1,2]. Following the legalization of cannabis use in Canada for medical and recreational purposes in 2018, commercial cultivation has expanded in all regions of the country. Correspondingly, reports of new and emerging pathogen occurrences have increased [1,2]. These pathogens are capable of infecting and destroying the roots, stems, foliage and inflorescences of cannabis plants grown under greenhouse conditions and indoor environments, as well as under field conditions [1,2,3,4,5,6,7,8,9,10,11,12,13,14]. The pathogen pressures are generally greater on greenhouse-grown plants, as they are spaced at high densities and the plants are frequently pruned and handled physically, practices that are known to spread many fungal, bacterial and viral pathogens [15]. The commonly used practice of the vegetative propagation of cannabis plants from cuttings derived from stock (mother) plants is also known to spread a number of pathogens [1,2,3,4]. Among the range of pathogens reported on cannabis plants, the emergence of hop latent viroid (HLVd), first recorded in 2019–2020 from the USA and Canada, has raised the most significant concern [16]. The rapid spread of the viroid has been shown to result in negative impacts on cannabis plant growth and quality [16]. For example, the viroid causes a reduction in the growth of stems, roots and inflorescences, resulting in stunted plants with poorly developed inflorescences [16]. Viroid infection also reduces the development of stalked glandular trichomes that are the sites of production and storage of a range of cannabinoids and terpene compounds [17,18]. Consequently, the economic impacts of HLVd are high, and the pathogen is estimated to have caused several billions of dollars in lost revenue to date in North America [19]. The losses due to HLVd in other parts of the world are unknown but are likely to be equally significant.

The range of symptoms of cannabis plants infected by HLVd has been recently described [16]. What remains unknown is how the viroid spreads within commercial cannabis production facilities and the extent to which it can survive once introduced. Viroids are the smallest group of pathogens known to affect plants, and are comprised solely of short RNA molecules, 226–430 nucleotides in length [20,21,22,23]. The viroids are totally dependent on their host plants for replication since their genetic material is insufficient to encode for any proteins. Various aspects of the biology of these unique pathogens can be found in several recent studies [24,25,26,27]. From the perspective of disease management, which is of significant importance to the cannabis industry, several approaches have been described to mitigate the economic impact of viroids on other susceptible agricultural crop species [28,29,30,31]. These include the destruction of diseased plants, the use of sanitizing chemicals to reduce survival and prevent spread, and meristem tip culture to provide pathogen-free planting material [28,29,30,31,32,33,34,35]. Regarding cannabis, several diagnostic methods have been described recently for HLVd detection (2). However, there have been few studies that provide insight into how the viroid can spread, the impact of sanitizing chemicals on viroid survival (based on the integrity of the RNA molecule that is detectable by PCR amplification), and other aspects of its biology in cannabis plants that can lead to a better understanding of the epidemiology and management of this devastating pathogen.

Therefore, the objectives of this study were to (i) conduct transmission studies to demonstrate the infection of cannabis plants by HLVd; (ii) study the potential mechanisms by which HLVd can spread within and between cannabis plants; (iii) determine the impact of viroid infection on trichome development; (iv) establish the role of pollen and seeds in transmission of HLVd; (v) study the longevity of HLVd in plant tissues under different environmental conditions; (vi) evaluate disinfectants and chemicals that can potentially denature HLVd RNA; and (vii) evaluate meristem tip culture for the potential to eliminate HLVd through tissue culture.

## 2. Results

### 2.1. Detection of HLVd in Stock Plants and in Flowering Plants

**Figure 1:**
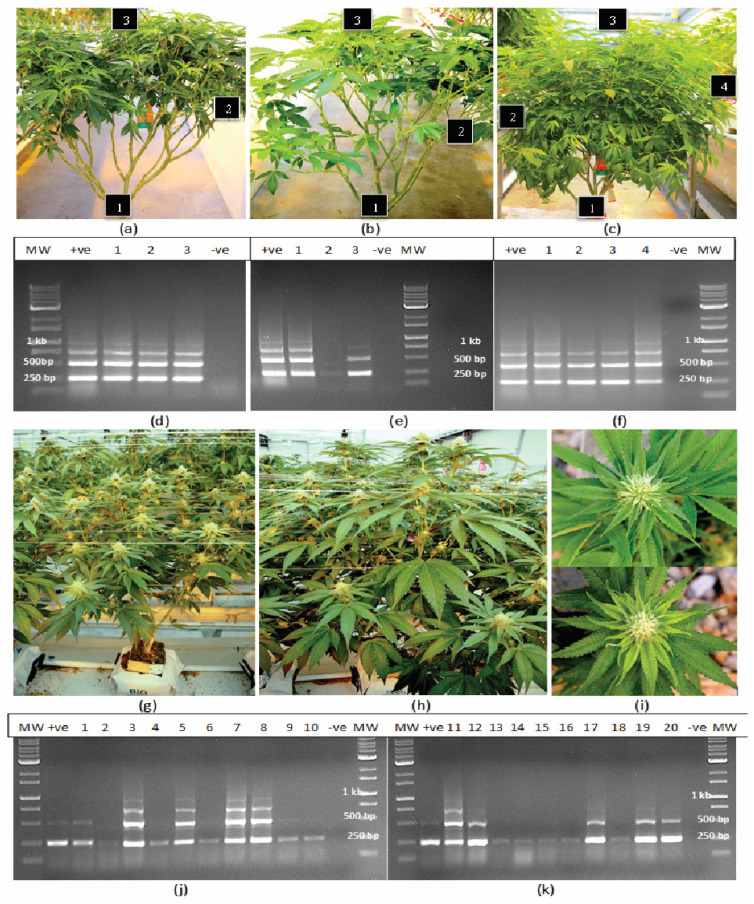
Sampling of different tissues from cannabis stock and flowering plants for hop latent viroid. (**a**–**c**) Stock plants of three genotypes were sampled at positions (labelled 1–4) corresponding to roots and leaves from the bottom, middle and top of the canopy. (**d**–**f**) RNA banding patterns following RT-PCR of samples from the plants shown in (**a**–**c**). Multiple bands were observed in all samples except for a sample from the lower position of the plant in (**b**). The band sizes were approx. 256 bp, 512 bp and 768 bp. (**g**) Symptoms of overall stunted growth and reduced inflorescence development due to HLVd infection on a plant of genotype A5 in a flowering room containing trial plants. (**h**) A noninfected plant shows larger overall plant size and inflorescence development. (**i**) Comparison of inflorescence development in a noninfected plant (top) and an HLVd-infected plant (bottom) shows reduced overall size and chlorophyll development due to infection. (**j**,**k**) RT-PCR analysis of flowering stem tissues from 20 randomly selected symptomatic plants within a trial greenhouse compartment show that the presence of a 256 bp size fragment characteristic of HLVd in all plants, while 9 plants showed multiple banding patterns similar to those observed in stock plants.

Samples of leaf and root tissues obtained from stock plants of 10 cannabis genotypes grown in a designated facility (Figure 1a–c) were subjected to RT-PCR. The results for three genotypes are presented in Figure 1. They show the viroid was present in leaf samples from the bottom, middle and top of the plant, but not consistently for all genotypes. In some stock plants, the viroid was not detected in the bottom leaves, as shown in Figure 1e. In other plants, it was not detected in the upper leaves. The most consistent source of tissue in which HLVd was detected in all genotypes tested was the roots. Following RT-PCR, the gels consistently revealed the presence of three bands, with estimated sizes of 256 bp, 512 bp and 768 bp (Figure 1d–f). In some samples, a faint band at 924 bp could also be seen (Figure 1f). The bands were all confirmed to be HLVd through sequence analysis and comparison to various sequences retrieved from GenBank, with 100% sequence similarity.

In flowering plants of one genotype (A5), which was grown within a greenhouse compartment containing trial plants, symptoms of HLVd infection were quite apparent, with visibly stunted growth of affected plants and reduced size of the developing inflorescence stems (Figure 1g–i), symptoms that are consistently observed in HLVd-infected plants [16]. Samples of stem tissues from 20 randomly selected symptomatic plants showed that they all contained the 256 bp band, confirming the presence of HLVd. In addition, in nine of these plants, multiple bands similar to those seen in stock plants were also observed (Figure 1j,k).

### 2.2. Effect of Ribonuclease Enzymes on HLVd Integrity

**Figure 2:**
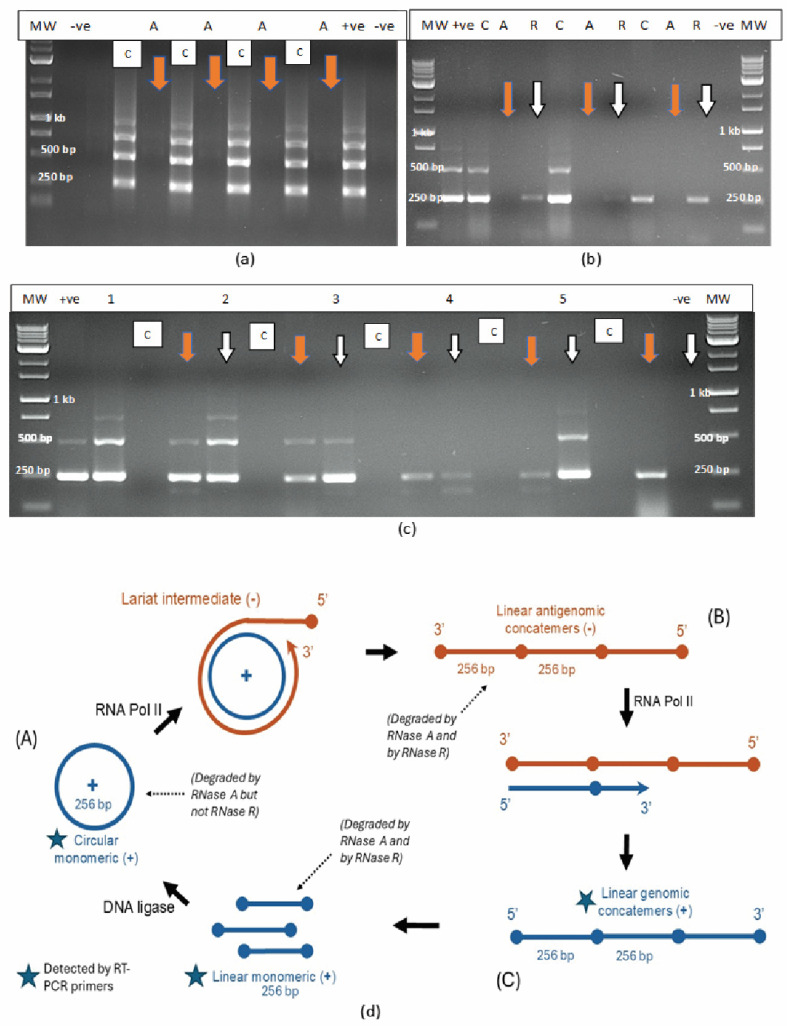
(**a**) RNA extracted from HLVd-infected plant tissues exposed to ribonuclease A (an endonuclease) followed by RT-PCR shows the complete digestion of the RNA in all lanes (marked by arrows). (**b**) RNA extracted from three infected cannabis genotypes exposed to ribonuclease A and ribonuclease R (an exonuclease) followed by RT-PCR shows complete digestion with ribonuclease A (brown arrows) and digestion of only the 512 bp fragment by ribonuclease R (white arrows). C = control samples without enzymes. (**c**) RNA extracted from five cannabis genotypes (labelled 1–5) exposed to ribonucleases A and R followed by RT-PCR shows complete digestion with ribonuclease A (brown arrows) and digestion of the 512 bp and 768 bp fragments by ribonuclease R (white arrows) for some genotypes. In genotypes 3 and 5, digested with ribonuclease R, the intensity of the 256 bp band was also reduced. C = control samples without enzymes. In all figures, MW = molecular weight standard, +ve = positive control sample infected with hop latent viroid, −ve = water control, A = ribonuclease A, R = ribonuclease R. (**d**) Schematic representation of the replication steps of the hop latent viroid RNA that are subjected to enzymatic degradation. Starting with the circular monomeric form (+ve strand, 256 bp) (A), replication using the host DNA-dependent RNA polymerase II creates a linear −ve strand (Lariat intermediate) to subsequently produce the linear antigenomic concatemers shown in (B). These are converted to linear genomic concatemers of varying sizes, which are digested by a (presumed) ribozyme to give rise to linear monomeric genomic (+) strands shown in (C). The results from enzyme digestion shown in (**a**–**c**) indicate that the circular monomeric form is not degraded by ribonuclease R while the linear concatemers are degraded by both ribonucleases A and R. In addition, the PCR primers detect the circular monomeric form as well as the linear genomic concatemers of varying sizes, giving rise to the multiple bands shown in Figure 1 and Figure 2 (adapted from McKernen and Helbert [36]).

The addition of laboratory-grade RNase A (1–2 units) to purified HLVd RNA extracted from leaf tissues of four cannabis genotypes followed by incubation for 10–20 min at room temperature (21–23 °C) resulted in total degradation and no bands were observed compared to the untreated control (Figure 2a), which showed four bands. By comparison, when these samples were treated with two units of RNase R, two outcomes were observed: the 512 bp band was digested (Figure 2b) or the 512 bp and 768 bp bands (where present) were partially digested and the 256 bp band was reduced in intensity (Figure 2c). These results demonstrate the differential sensitivity of the different-sized RNA bands of HLVd to the activity of RNase A (an endonuclease) and RNase R (an exonuclease) under similar experimental conditions. Based on these findings, together with those from previously published reports [21,23,25,27,36], the proposed replication steps for HLVd are shown in Figure 2d. The circular and linear monomeric forms of 256 bp size showed differential sensitivity to digestion by RNase R, while both the circular and the linear multimeric concatemers forms were digested by RNase A. By adding the enzymes separately to each sample, the circular 256 bp monomeric form could be distinguished from the linear concatemers seen on the RT-PCR gels. The 256 bp bands, however, could comprise a combination of circular and linear forms which could not be distinguished.

The secondary structures predicted from the full 256 bp sequence from cannabis genotype ‘M1’ (GenBank accession OQ420426) of hop latent viroid as depicted by mfold [37] are shown in Supplementary Figure S1. The complementary base-pairing and stem loop structures that produce a rod-like shape were determined by the programme algorithm, which calculates the most thermodynamically stable configuration of an RNA sequence by predicting how suboptimal folding may occur and how bases could pair up to minimize free energy. The proposed structures show the complementary base-pairing and the stem loop structures, as well as the secondary structure of the RNA molecule based on different Δ*G* values (Supplementary Figure S1).

### 2.3. Transmission of HLVd Through Cuttings from Infected Stock Plants

**Figure 3:**
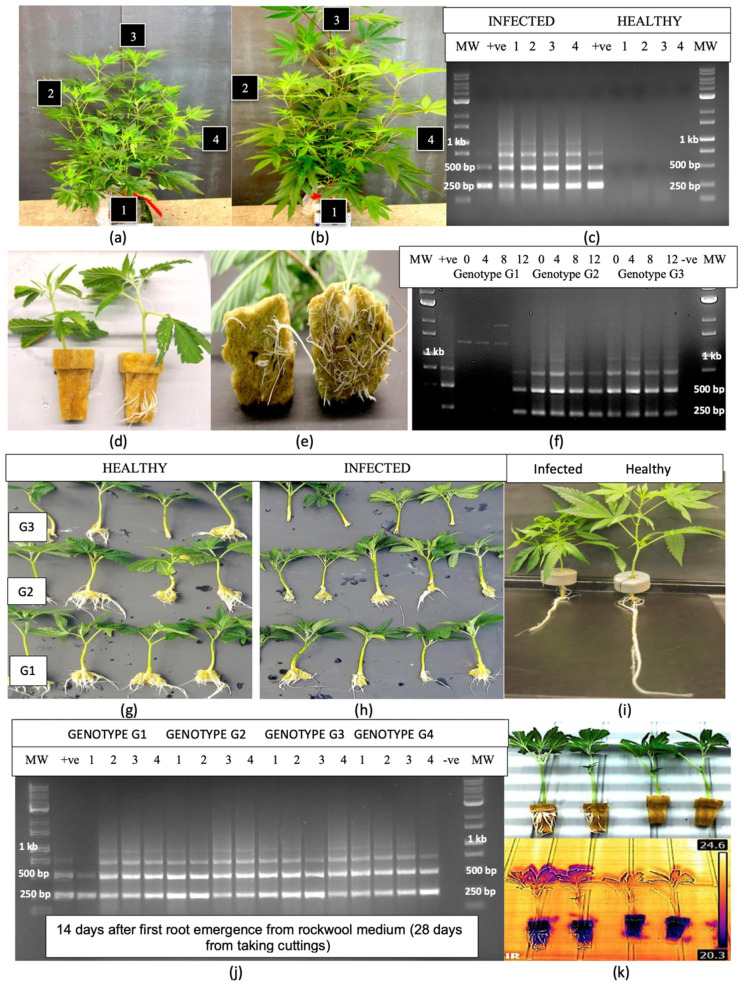
Transmission of hop latent viroid through vegetative cuttings originating from infected stock plants of several cannabis genotypes. (**a**,**b**) Symptomatic and healthy-appearing plants, respectively, of genotype ‘S3’ were sampled at positions marked by 1–4. Symptoms of stunting and shorter internode length are shown in (**a**). (**c**) Banding patterns following RT-PCR of the plants shown in (**a**,**b**) confirms the viroid was present in all tissue samples taken from (**a**) but was absent in all samples taken from (**b**). (**d**,**e**) The extent of root emergence from infected and healthy cuttings in rockwool blocks. On the left side in each photo, cuttings were taken from an infected plant compared to a healthy plant (right). (**f**) Detection of hop latent viroid in roots of cuttings of three cannabis genotypes (G1, G2, G3) infected with HLVd in which the roots had just emerged from the rockwool blocks (time 0) and at various times subsequently (4, 8, 12 days). In genotype G1, the viroid was only detected at day 12 compared to all sampling times in genotypes 2 and 3, suggesting a slower movement of the viroid into the roots had occurred. (**g**,**h**) Root development on cuttings of each of three genotypes shown in (**f**) at 14 days after emergence from the rockwool blocks compared to cuttings from HLVd-infected stock plants (**h**). (**i**) Growth comparison of a cutting from an infected stock plant (left) of genotype ‘G1’ compared to a healthy plant (right) at 3 weeks. (**j**) Banding patterns following RT-PCR of the cuttings shown in (**h**) plus genotype G4, at 14 days after first emergence of roots from the rockwool blocks. All cuttings contained the viroid. In all figures, MW = molecular weight standard; +ve = positive control sample infected with hop latent viroid; −ve = water control. (**k**) Infra-red image analysis conducted on four cannabis cuttings, two rooted and two unrooted (top), shows a higher temperature emittance in the rooted cuttings (24.6 °C) compared to the unrooted cuttings (20.3 °C) (bottom). All four cuttings were positive for HLVd, suggesting the IR image was reflecting greater transpiration from rooted cuttings and not the presence/absence of HLVd.

Stock plants of genotype ‘S3’ with distinct symptoms of stunting, reduced internode length and reduced leaf size (Figure 3a) were sampled at four positions (roots, bottom leaf, middle leaf and top leaf) and compared to a control plant not displaying these symptoms (Figure 3b). The banding patterns on the RT-PCR gels from these tissue samples (Figure 3c) were identical to those shown in Figure 1, consisting of four bands of varying sizes that were absent in the asymptomatic plant. When cuttings obtained from this plant were rooted in rockwool blocks, the emergence of roots and root length were markedly reduced in the HLVd-positive cuttings compared to the HLVd-negative cuttings (Figure 3d,e). Root samples taken at 0, 4, 8 and 12 days following the first root emergence from rockwool blocks of three additional cannabis genotypes (‘T8’, ‘M1’ and ‘B2’) all displayed identical banding patterns consistent with the presence of HLVd. However, in genotype ‘T8’, these bands were only observed at day 12 of root emergence, reflecting a delayed detection of the viroid (Figure 3f). The appearance of the roots on cuttings of the latter three genotypes is shown in Figure 3g,h. Consistently, all cuttings from HLVd-infected plants showed reduced root emergence and root development (Figure 3g) when compared to the non-infected controls (Figure 3h). On genotype ‘S3’, a pronounced reduction in root development and plant growth resulting from HLVd infection is shown in Figure 3i. At 14 days after root emergence (28 days after cuttings were obtained), the roots of all cuttings originating from the infected stock plants of four cannabis genotypes (which included the three genotypes shown in Figure 3f) consistently showed the multiple banding pattern characteristic of HLVd replication (Figure 3j). When a comparison of the infra-red (IR) spectrum of rooted and unrooted cuttings was made (Figure 3k), a clear distinction was observed, as the unrooted cuttings displaying a higher leaf surface temperature (24.6 °C) compared to the rooted cuttings (20.3 °C). However, since both sets of cuttings were subsequently found to be HLVd-positive, the IR method was not identifying diseased plants but rather those with a reduced rooting frequency that could also have been due to other causes.

### 2.4. Transmission of HLVd Through Roots of Infected Plants

#### 2.4.1. Kloner Experiments

**Figure 4:**
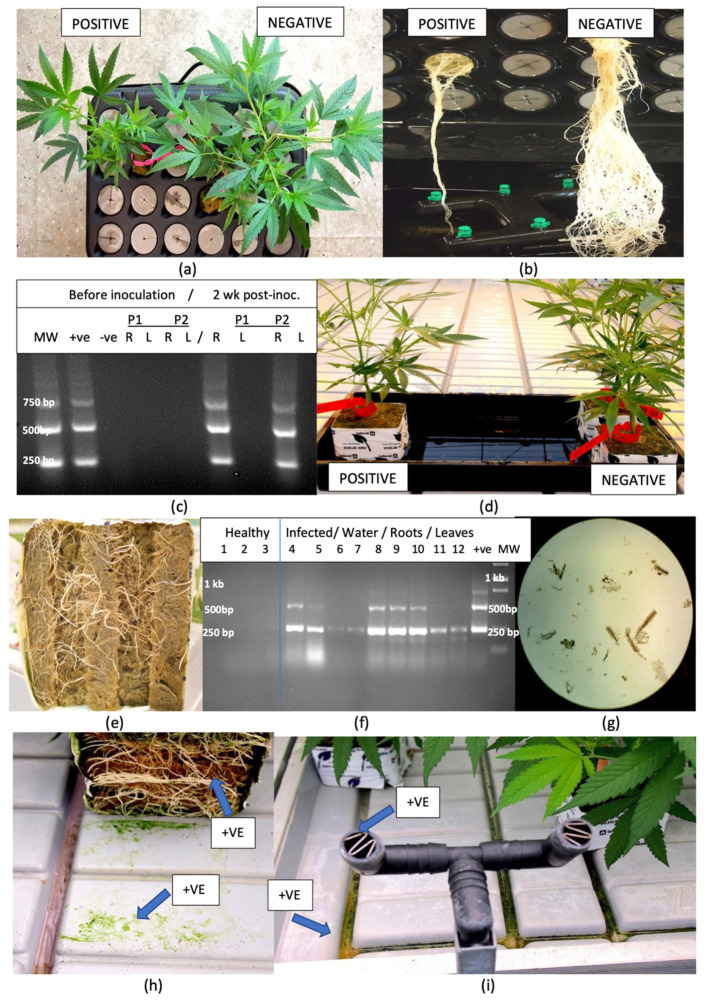
Transmission of hop latent viroid through infected roots of cannabis plants and through recirculated nutrient solution. (**a**) Placement of a HLVd-infected cutting and a healthy cutting of genotype ‘B2’ in the same Kloner. (**b**) Root development on both plants at 2 weeks shows a difference between the infected and healthy plant. (**c**) RT-PCR analysis of plants from the Kloner before and after the start of the experiment. Results are shown for duplicate plants (P1, P2). No bands were observed in the healthy plants before inoculation and bands reflecting positive detection of HLVd were observed in the roots of these plants after 2 weeks. (**d**) Placement of a HLVd-infected plant of ‘B2’ with healthy plants at the opposite ends of a tray. The trays were flooded twice daily for 2 weeks, after which plants were sampled. (**e**) Underside of the rockwool block showing development of roots in the infected ‘B2’ plant used in the experiment. (**f**) RT-PCR results from analysis of healthy and infected plants used in the experiment, water (nutrient) sample, and roots and leaves from the previously healthy plants which now contain the viroid. (**g**) A sample of nutrient solution from around the root zone shows the presence of small broken roots and sloughed off root cells. (**h**,**i**) Detection of HLVd (as denoted by +ve) using RT-qPCR. The viroid was present on the root surface, on the surface of the table immediately below the roots, in the grooves of the table where irrigation water accumulated, in drainage water and in the irrigation nozzles releasing recirculated nutrient solution.

The placement of an HLVd-positive rooted cutting of cannabis genotype ‘B2’ showing distinct symptoms of stunting and reduced root growth (Figure 4a,b) next to an HLVd-negative cutting with large leaves and extensive root development within a Kloner with recirculating nutrient solution for a period of 2 weeks resulted in the transmission of the viroid to the previously healthy plant (Figure 4c). The RT-PCR banding pattern characteristic for HLVd was observed in two replicate samples taken from the previously uninfected plant of ‘B2’. The experiment was repeated with cannabis genotype ‘K4’ with similar results.

#### 2.4.2. Greenhouse Experiments

HLVd-infected plants of ‘S3’ and ‘B2’ were placed on a tray distal to asymptomatic healthy plants of the same genotypes for a 2-week period and subjected to daily flooding (Figure 4d). The cuttings were initiated in rockwool blocks and the emerging roots were visible on the bottom (Figure 4e). In three healthy plants, no bands were obtained following RT-PCR analysis of root samples at the start of the experiment (Figure 4f). By comparison, root samples from two infected plants at the start of the experiment confirmed the presence of HLVd. Water samples taken from the flooding treatment showed faint bands of 256 bp size that corresponded to HLVd (Figure 4f, lanes 6,7). A microscopic examination of these water samples showed they contained broken root segments and sloughed-off roots cells (Figure 4g). Subsequently, root and leaf samples from the previously healthy ‘B2’ plants subjected to flooding showed the presence of HLVd in these tissues after two weeks of exposure to water, confirming the transmission of the viroid (Figure 4f, lanes 8–12). The experiment was repeated once with similar results.

### 2.5. Distribution of HLVd in a Trial Greenhouse Environment

Samples of crushed roots and swabs obtained from the surface of roots of symptomatic plants presumed to be infected with HLVd in a trial greenhouse, as well as swabs taken of the surface of propagation tables, irrigation nozzles, and samples of pooled water on tables and recirculated nutrient solution, were assayed for the presence of HLVd using RT-qPCR. The results from duplicate samples are presented in Table 1. At least one sample from each location was positive for HLVd, with low Ct values (reflecting higher viroid levels) observed in crushed roots and in swabs taken from the surface of tables underneath the rockwool blocks (Figure 4h,i). Samples below a Ct value of 33 were considered positive for HLVd (Table 1). To correlate these Ct values to approximate viroid copy numbers, a dilution curve was established using synthetic viroid clones of known concentrations of HLVd (Supplementary Figure S2). A series of control dilutions was made using synthetic HLVd in nuclease-free water and subjected to the same PCR conditions as the samples that were mixed with plant tissues. These latter samples were used to determine the limits of detection by adding 100 μL of each serial dilution to 900 μL of homogenized tissue (100 mg of stem tissues) from an HLVd-negative plant, followed by PCR. The results showed that approximately 100 to 10,000 copies of HLVd were present on the surface of greenhouse benches. The limit of detection was determined at a Ct value of 33, or 10 copies of the viroid.

### 2.6. Transmission of HLVd by Mechanical Inoculation

**Figure 5:**
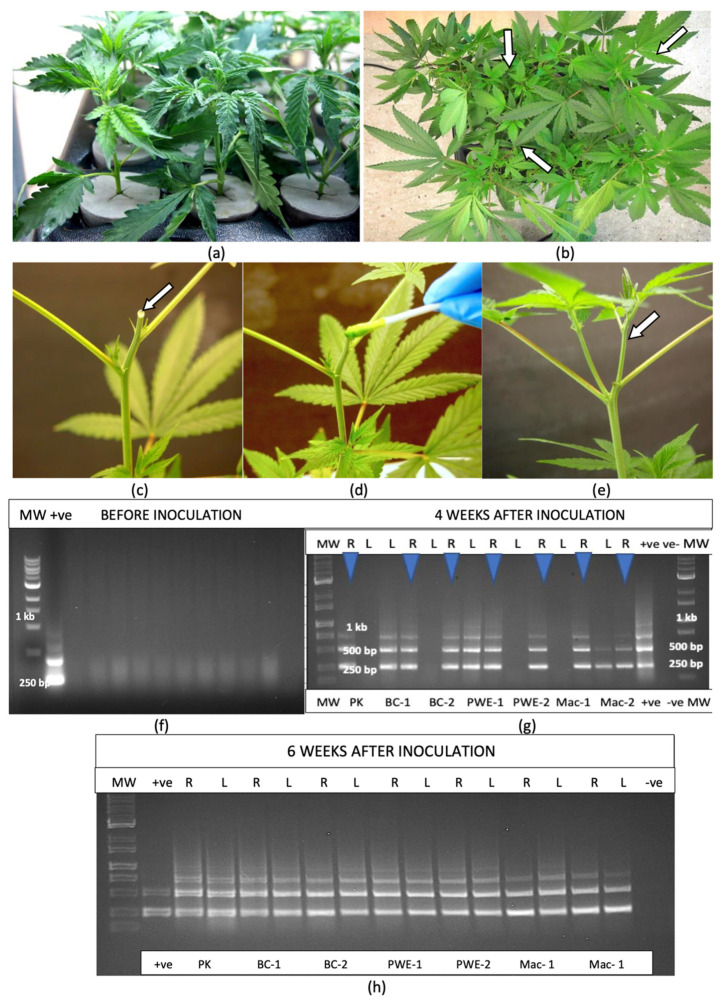
Transmission of hop latent viroid through stem inoculations conducted on hydroponically grown plants in Kloners. (**a**) Individual cuttings are inserted into Styrofoam holders and held in place and allowed to root. (**b**) After 4 weeks of growth, individuals stems on each plant (shown by arrows) were cut and discarded. (**c**) The exposed cut stem surface (arrow) with a drop of exudate. (**d**) Sap extracted from leaves of an HLVd-infected plant was dabbed onto the exposed surface of the cut stem using a Q-tip. (**e**) Two weeks after inoculation, new shoot growth can be seen on either side of the cut stem (arrow). (**f**) Results from RT-PCR of stem cuttings used in the experiment, showing the absence of HLVd in all cuttings used. (**g**) RT-PCR conducted on root (R) and leaf (L) tissues of four genotypes inoculated 4 weeks prior with HLVd shows the presence of the viroid in all root samples (blue arrows) and in three out of seven leaf samples. Multiple bands are indicative of active replication of the viroid. (**h**) Results from RT-PCR conducted 6 weeks after stem inoculation of four genotypes of cannabis (PK, BC, PWE, Mac) shows the presence of the viroid in all tissue samples (roots and leaves).

Inoculations were made of rooted cuttings of five cannabis genotypes that were grown in Kloners for 4 weeks under 24 h of light (Figure 5a,b) by exposing the surface of a cut stem to infectious sap (Figure 5c–e) followed by sampling for HLVd at 4 weeks and 6 weeks post-inoculation. RT-PCR analysis of four replicate samples showed that plants were free of HLVd at the start of the experiment (Figure 5f). Multiple banding patterns characteristic of HLVd were observed in the inoculated plants (Figure 5g).

At 4 weeks after inoculation, all root and some leaf samples (four out of seven) from four genotypes, with from two to four replicates of each, were positive for HLVd (Figure 5g). After 6 weeks, consistent banding patterns were observed in all leaf and root samples (Figure 5h), indicating successful transmission of HLVd had occurred in all four genotypes.

**Figure 6:**
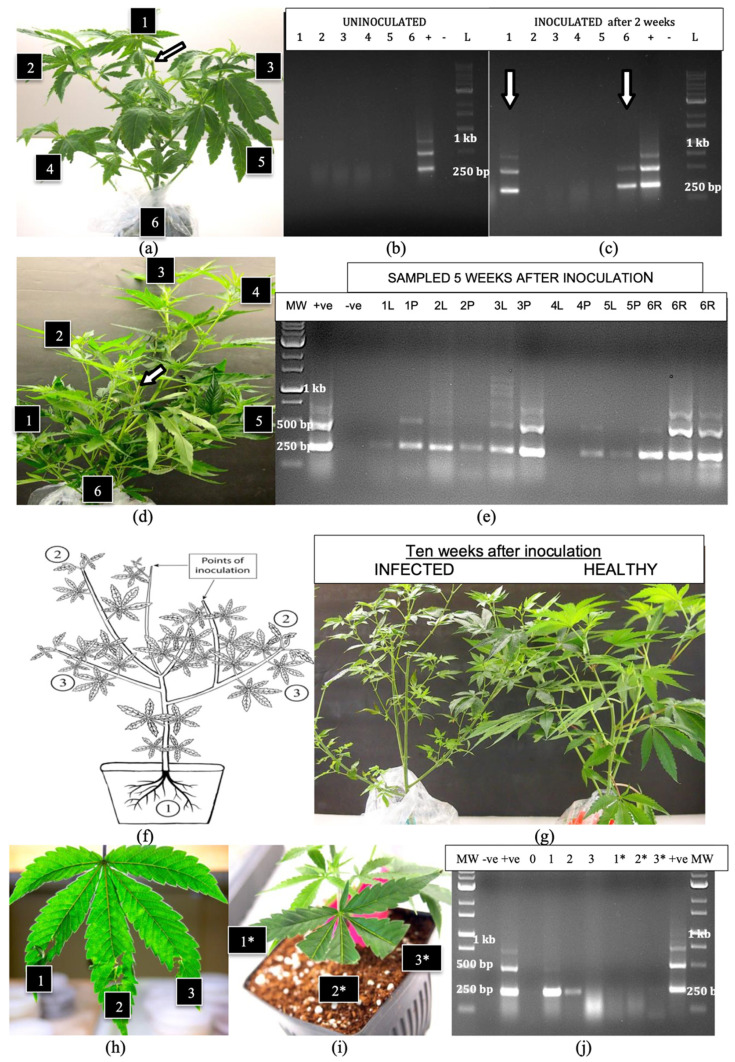
(**a**) Inoculation of a 6-week-old plant of genotype ‘S3’ on a cut stem surface (arrow) shows the locations (1–6) where samples were taken prior to inoculation and after 2 weeks. (**b**) RT-PCR of tissues sampled before inoculation shows the plant was HLVd-negative. (**c**) RT-PCR of tissues sampled 2 weeks after inoculation shows HLVd is present in the youngest leaves (1) and in the roots (6). (**d**) The same plant shown in (**a**) was sampled 5 weeks after inoculation at positions (1–6). (**e**) RT-PCR analysis shows the viroid was present at various sampling positions in the foliage and in the roots. The multiple bands characteristic of HLVd can be seen. In many samples, petiole tissues showed more intense bands compared to leaf tissues. (**f**) Schematic representation of the spread of HLVd from stem inoculations to various parts of the plant, with detection in the roots (1) followed by detection in young leaves (2) and then in the middle and lower leaves (3). (**g**) Symptoms on a plant inoculated on a cut stem surface and grown for 10 weeks (left) compared to an uninoculated control plant (right). The leaves are reduced in size and the overall growth is less vigorous. (**h**) Physical abrasion of leaves that resulted in tearing at positions marked (1, 2, 3) followed by HLVd sap inoculation was compared to the cutting of leaf edges with scissors shown in (**i**), and then sap inoculated at positions marked (1*, 2*, 3*). (**j**) RT-PCR analysis of leaves from inoculated plants wounded as shown in (**h**) revealed the presence of HLVd in three leaf samples while plants wounded as shown in (**i**) did not become infected, as shown by the absence of bands corresponding to HLVd.

Additional inoculations were made on larger plants (6 weeks of age) of genotypes ‘S3’ and ‘B2’ on cut stem surfaces (Figure 6a) and these plants were grown for an additional 5 weeks under 24 h of light. Root, leaf and petiole samples taken from various positions around the plants at 2 and 5 weeks after inoculation were subjected to RT-PCR. At 2 weeks, the viroid was absent in the roots and leaves of uninoculated plants (Figure 6b) but was detected in the roots and in the uppermost leaves of inoculated plants (Figure 6c), and was absent in other parts of the plant. At 5 weeks after inoculation, when the plants had grown considerably larger (Figure 6d), HLVd was detected in all root and petiole samples, but was absent in one leaf sample taken from the top of the plant (position 4) and was faintly detected in one leaf sample taken from the bottom of the plant (position 5) (Figure 6e). The multiple banding pattern characteristic of HLVd was observed in all tissues. In most samples, petiole tissues showed more intense bands compared to leaf tissues, suggesting a higher accumulation of the viroid. A schematic representation of the spread of HLVd following stem inoculation is shown in Figure 6f. The viroid was first detected in the roots, followed by spread to the upper leaves and then to the lower leaves of the plant. At 10 weeks after inoculation, these plants developed symptoms of HLVd infection, which included detection in the roots, followed by spread to the upper leaves and then the lower leaves of the plant. At 10 weeks after inoculation, these plants developed symptoms of HLVd infection, which included smaller leaves and less vigorous growth (Figure 6g). The mechanical inoculation of leaves of cannabis cuttings that included exposing physically macerated leaf lamina to infectious sap (Figure 6h) or introducing sap to the cut ends of leaves (Figure 6i) showed transmission in the former but not the latter (Figure 6j).

### 2.7. Effect of a 12:12 H Photoperiod on Viroid Spread

**Figure 7:**
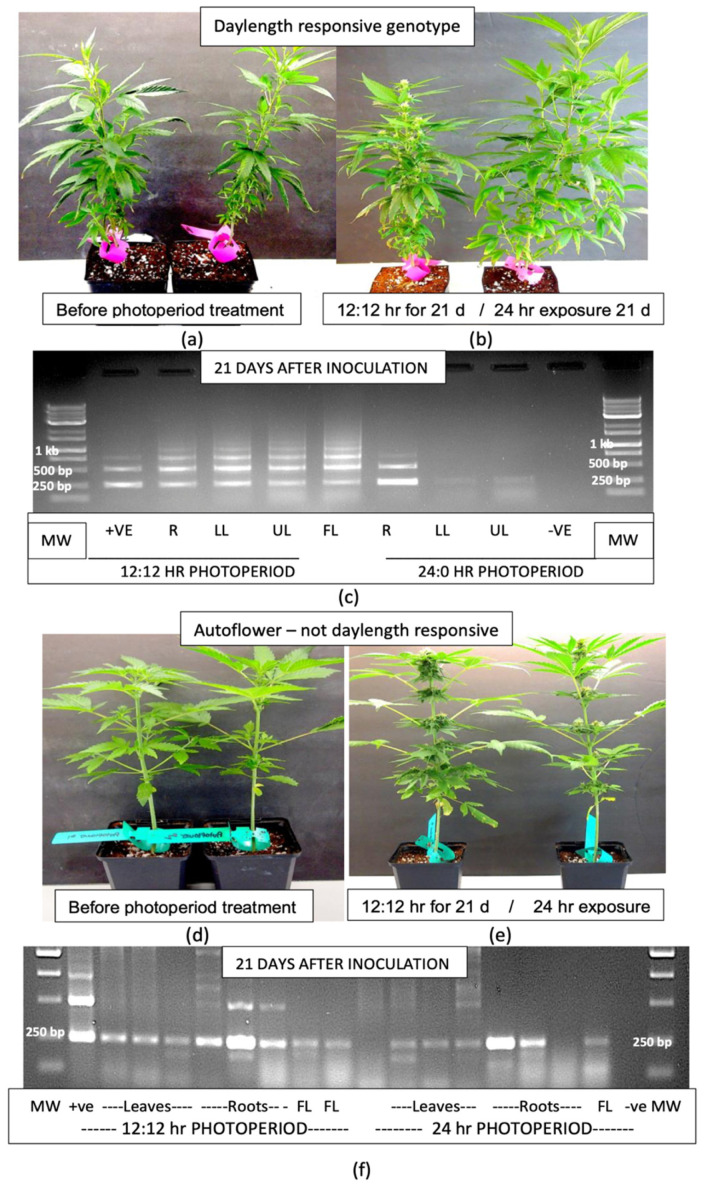
The transfer of one set of 4-week-old HLVd-positive plants of genotype ‘H6’ displaying no symptoms of infection (**a**) from a 24 h photoperiod to a 12:12 h photoperiod in a growth chamber promoted inflorescence development when compared to plants maintained under constant 24 h light (**b**). The flowering plants were shorter in stature and more compact than the vegetative plants. When the experiment was repeated using artificially inoculated plants of genotypes ‘K4’, ‘M1’ and ‘S3’, inoculated 24 h prior to the transfer to a 12:12 h photoperiod, a similar transition to flowering was observed. When the roots, lower leaves, mid-leaves and flowers were sampled 21 days later and compared to plants kept under constant 24 h light for HLVd presence, the difference was significant (**c**). All tissues sampled from the plants grown under a 12:12 h photoperiod showed the characteristic multiple banding pattern for HLVd, while those with 24 h light showed the viroid was present in the roots, while faint bands could be seen in the leaves (**c**). RT-qPCR data from samples taken at 12 and 21 days following transfer to the different photoperiods for genotypes ‘M1’, ‘K4’ and ‘S3’ are shown in Table 2. When the inoculation experiment was repeated with an autoflower genotype, in which the plants produce inflorescences at a certain growth stage regardless of photoperiod, the transition from vegetative growth (**d**) to flowering was observed in both sets of plants—those placed under the 12:12 h photoperiod and constant 24 h light (**e**). The former plants visually appeared to have a slightly more advanced inflorescence development. The RT-PCR results from tissues taken from these two sets of plants showed similar banding patterns, with a predominant band of 256 bp size observed in all tissues (roots, leaves, flowers) and at both photoperiods (12:12 and 24 h). The root samples had a more intense band overall compared to the rest of the plant (**f**).

The transfer of one group of 4-week-old plants of genotype ‘H6’ displaying no symptoms of infection but with positive detection of HLVd in the roots (Figure 7a) from a 24 h photoperiod to a 12:12 h photoperiod in a growth chamber induced inflorescence development when compared to plants that were maintained under constant 24 h light (Figure 7b). The infected flowering plants were shorter in stature and more compact than the infected vegetative plants. Plants of genotypes ‘K4’, ‘M1’ and ‘S3’, grown under 24 h of light and then stem-inoculated 24 h prior to transfer to a 12:12 h photoperiod, showed a similar transition to flowering. Root, lower leaf, mid-leaf and flower samples taken 21 days later were compared for HLVd presence to those of plants similarly inoculated but kept under constant 24 h light. The plants grown under a 12:12 h photoperiod showed the characteristic multiple banding pattern for HLVd in all tissues sampled (Figure 7c), while those under the 24 h light regime showed viroid presence in the roots, with faint bands in the upper leaves after 3 weeks (Figure 7c). RT-qPCR data from samples taken at 12 and 21 days following transfer to the different photoperiods for genotypes ‘M1’, ‘K4’ and ‘S3’ are shown in Table 2. In all tissues sampled from the plants grown under a 12:12 h photoperiod, the Ct values were lower (viroid levels were higher) in the three genotypes compared to constant 24 h light (Table 2). When comparing tissue types, the top leaves had the highest viroid levels compared to mid-leaves and roots. Where flowers were present (in the 12:12 h photoperiod), viroid levels were the highest in these tissues compared to all other tissues (Table 2). There were also differences between the genotypes in the levels of viroid present, with genotype ‘S3’ (considered to be highly susceptible to HLVd) showing the highest levels, followed by genotype ‘M1’ (highly susceptible) and lastly genotype ‘K4’ (moderately susceptible). However, since these represent single Ct values from nonreplicated samples, they infer trends of genotype susceptibility without statistical analysis.

When the inoculation experiment was repeated with an autoflower genotype, in which the plants produce inflorescences at a certain growth stage regardless of the photoperiod, the transition from vegetative growth (Figure 7d) to flowering occurred in both sets of plants—those placed under the 12:12 h photoperiod as well as those under constant 24 h light (Figure 7e). The former plants appeared to be slightly more advanced in their inflorescence development than the latter. The RT-PCR results from tissues sampled 3 weeks after inoculation from these two groups of plants showed similar banding patterns (Figure 7f). A predominant band of 256 bp size was observed in all tissues (roots, leaves, flowers) and at both photoperiods (12:12 and 24 h). The root samples had multiple bands with greater general intensity compared to the remainder of the samples (Figure 7f), especially in the plants grown under a 12:12 h photoperiod. RT-qPCR analyses were not conducted on these plants.

### 2.8. Evaluation of Host Range of HLVd

**Figure 8:**
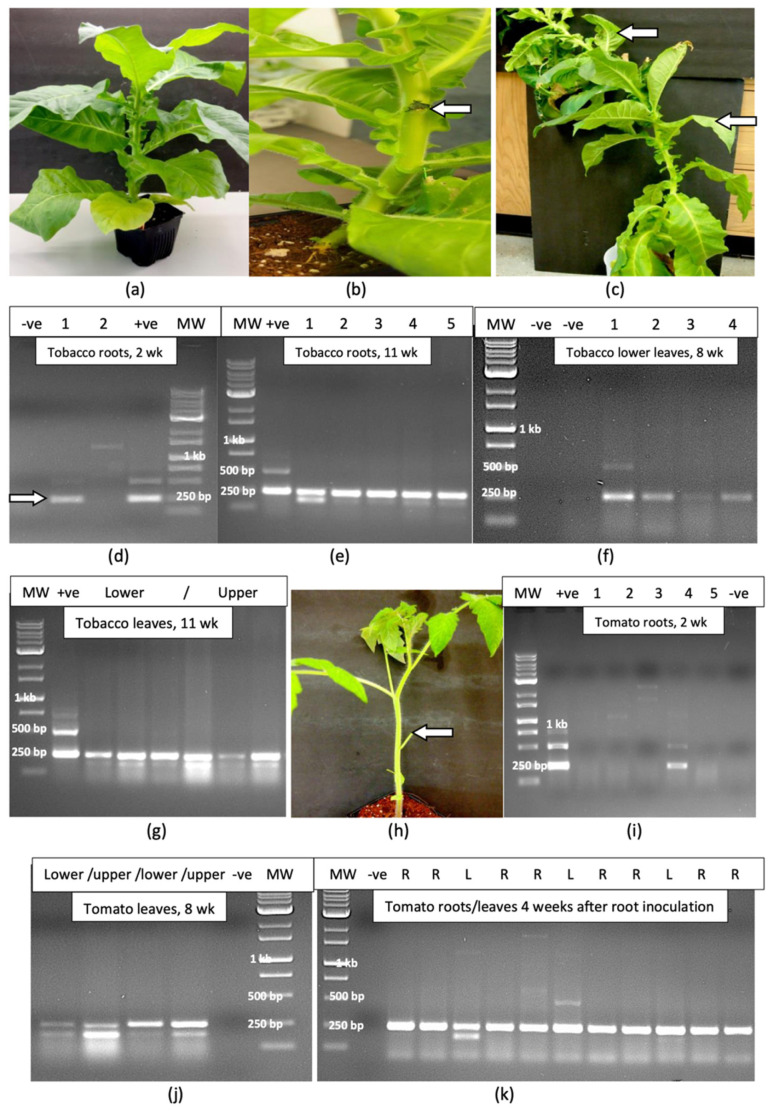
Artificial inoculation experiments conducted on tobacco (*Nicotiana tabacum* ’Samsun’) and tomato (*Solanum lycopersicum* ‘Celebrity’) plants. (**a**) Two-month old tobacco plant used for inoculations. (**b**) Infectious sap containing HLVd was introduced at the wound site where a leaf was removed (arrow). (**c**) Eleven week-old tobacco plant inoculated with HLVd was sampled at the points marked by arrows. (**d**) Detection of HLVd in the roots of one tobacco plant 2 weeks after inoculation at a petiole wound site as shown by the presence of a 256 bp band (arrow) that was absent in control plants. (**e**) Detection of HLVd in the roots of five tobacco plant 11 weeks after inoculation at a petiole wound site as shown by the presence of a 256 bp band. (**f**) Detection of HLVd in the lower leaves of four tobacco plants 8 weeks after inoculation at a petiole wound site as shown by the presence of a 256 bp band that was absent in control plants. (**g**) Detection of HLVd in the lower and upper leaves of three tobacco plants 11 weeks after inoculation at a petiole wound site as shown by the presence of a 256 bp band. (**h**) One month-old tomato plant used for inoculations showing the site of inoculation at a cut petiole (arrow). (**i**) Detection of HLVd in the roots of one out of five tomato plants 2 weeks after inoculation at a petiole wound site as shown by the presence of a 256 bp band that was absent in control plants. (**j**) Detection of HLVd in the lower and upper leaves of two tomato plants 8 weeks after inoculation at a petiole wound site as shown by the presence of a 256 bp band that was absent in control plants. (**k**) Detection of HLVd in the roots and lower leaves of four tomato plants 4 weeks after inoculation of the root system as shown by the presence of 256 bp bands.

The inoculation of tobacco plants at the exposed site where a leaf had been removed (Figure 8a,b) was followed by the sampling of root and leaf tissues for a period of up to 11 weeks, at which time the plants had reached a height of over 1.2 m (Figure 8c). At 2 weeks after inoculation, one plant out of five showed the presence of a 256 bp band in the root tissues (Figure 8d). At 11 weeks, all inoculated plants were positive for HLVd in the roots (Figure 8e). Uninoculated control plants did not show a band corresponding to HLVd. The viroid was detected in the lower leaves of four out of five inoculated plants after 8 weeks (Figure 8f) and in all lower and upper leaves of all inoculated plants at 11 weeks (Figure 8g). In all tobacco plants, only the 256 bp band was observed and the higher-molecular-weight fragments seen in cannabis tissues were absent. The band was confirmed to be that of HLVd through sequence analysis.

**Figure 9:**
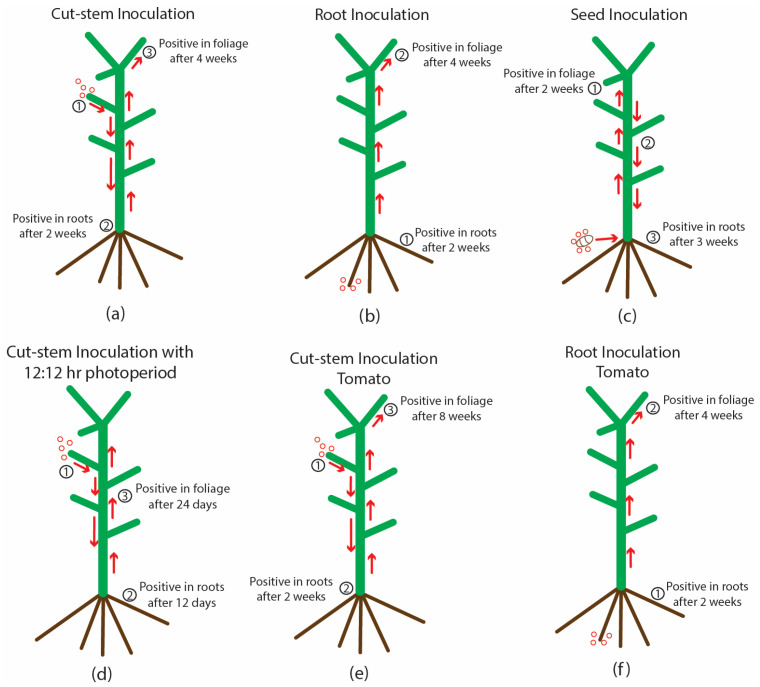
Schematic representation of the spread of hop latent viroid (red arrows reflect the direction of movement from 1 to 2 to 3) following different inoculation methods. (**a**) Cut-stem inoculation shows a downward movement of the viroid into the roots of inoculated cannabis plants within 2 weeks and subsequent detection in the foliage after 4 weeks. (**b**) Root inoculation shows detection of the viroid in the roots after 2 weeks and subsequent detection in the foliage after 4 weeks. (**c**) Seed inoculations shows detection of the viroid in the foliage after 2 weeks and detection in the roots after 3 weeks. (**d**) Cut-stem inoculation followed by exposure to a 12:12 h photoperiod shows detection in the roots after 12 days and in the foliage after 24 days. (**e**) Cut-stem inoculation of tomato plants shows detection in the roots after 2 weeks and in the foliage after 8 weeks. (**f**) Root inoculation of tomato plants shows detection in the roots after 2 weeks and in the foliage after 4 weeks.

On tomato plants that were inoculated on the cut surface of a petiole (Figure 8h), HLVd was detected in the roots of one plant out of five after 2 weeks (Figure 8i), and in all lower and upper leaves of all plants after 8 weeks (Figure 8j). In plants that were inoculated by applying infectious sap to the root system, the viroid was present in the roots and leaves of all plants after 4 weeks (Figure 8k). All uninoculated control plants were negative for HLVd. A summary of the spread of HLVd in cannabis and tomato plants following stem and root inoculation at various time periods is shown in Figure 9. The presumed direction of movement of the viroid in the inoculated plants is indicated. In these plants, HLVd was first detected in the roots, followed by the foliage. From inoculated cannabis seeds, the viroid was first detected in the leaves, followed by the roots. In plants in which the viroid was introduced through the roots, the spread to the foliage was much faster in both cannabis and tomato plants compared to stem inoculation (Figure 9). None of the tobacco or tomato plants shown to be infected by HLVd displayed any symptoms of infection when compared to noninfected control plants.

### 2.9. Assessing Flower Tissues, Trichomes and Resin for HLVd Presence

**Figure 10:**
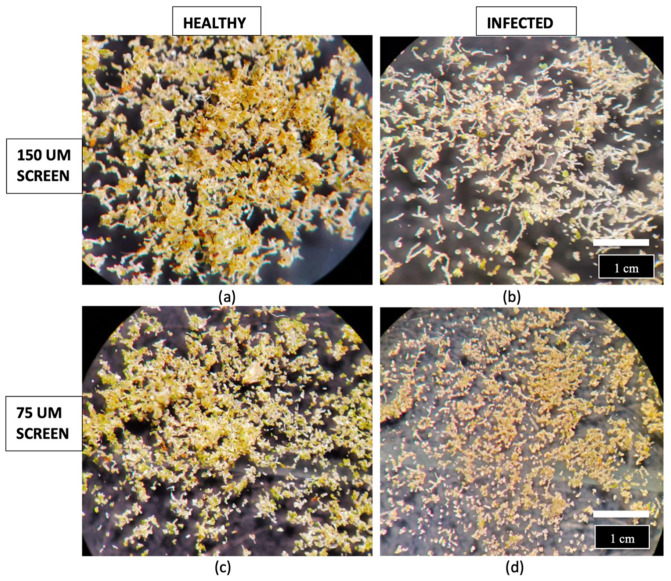
Impact of hop latent viroid infection on cannabis trichome development and size. (**a**) A healthy plant of genotype ‘D7’ produces large quantities of yellowish-brown trichomes collected on screen sizes of 150 µm (**a**) and 75 µm (**c**). An HLVd-infected plant produces fewer trichomes collected on the 150 µm screen that are yellowish-white in colour (**b**) and large quantities of smaller, poorly developed trichomes collected on the 75 µm screen (**d**). These images show a direct negative impact of HLVd infection on trichome development in this cannabis genotype (highly susceptible).

Dried inflorescence tissues from HLVd-positive and HLVd-negative flowering plants of genotypes ‘C9’ and ‘D7’ were gently crushed by hand and passed through a series of stacked sieves with penultimate mesh sizes of 150 µm and 75 µm. The volume of trichomes collected on the 150 µm screen was visibly greater from healthy compared to viroid-infected inflorescences (Figure 10a,b. Similarly, on the 75 µm screen, the volume of trichomes was greater from the healthy compared to infected plants (Figure 10c,d). From infected plants, the trichomes were smaller and appeared underdeveloped and were lighter in colour (Figure 10b,d), showing a visible negative impact of HLVd infection on trichome development.

**Figure 11:**
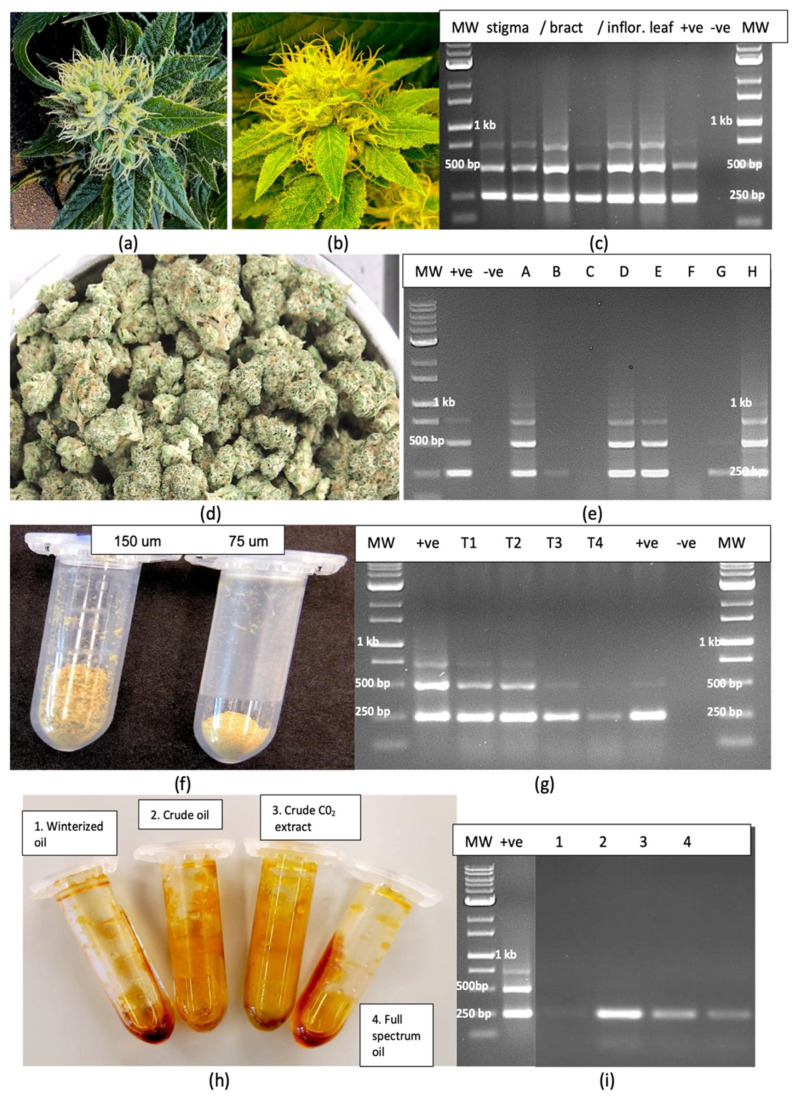
Detection of hop latent viroid in inflorescences of cannabis plants. (**a**) Healthy inflorescence compared to an HLVd-infected one (**b**) shows yellowing of the bract tissues due to the viroid. (**c**) Positive detection of HLVd via RT-PCR in the stigmatic tissues, bracts and inflorescence leaves from an infected plant. (**d**) Samples of dried inflorescences were subjected to RT-PCR, showing that 5 out of 8 were infected by HLVd (**e**). (**f**) Total trichome preparations from an infected cannabis inflorescence shows the fraction collected on a 150 µm screen and a 75 µm screen (**g**) RT-PCR analysis of trichome preparations shows the presence of HLVd in both the 150 um (T1, T2) and 75 µm fractions (T3, T4). (**h**) Resins extracted from trichomes of cannabis using four different methods. (**i**) RT-PCR analysis shows the presence of HLVd in three out of four trichome preparations. An extremely faint band can be seen in sample 1 (winterized oil). In all figures, MW = molecular weight standard; +ve = positive control sample infected with hop latent viroid; −ve = water control.

Inflorescence and trichome samples from HLVd-positive plants were also analyzed by RT-PCR. In symptomatic inflorescences on which symptoms of yellowing and stunting were observed compared to green inflorescences on healthy plants (Figure 11a,b), HLVd was detected in the stigmatic tissues, in excised bracts and in inflorescence leaves (Figure 11c), with multiple banding patterns characteristic of HLVd observed in all tissues sampled. These bands were absent in healthy inflorescence tissues. In dried flower tissues, HLVd was present in five out of eight samples (Figure 11d,e). The trichome preparation collected from sieving through both the 150 and 75 µm screens is shown in Figure 11f. In duplicate samples of each source, HLVd was observed to be present in the trichomes from infected plants via RT-PCR (Figure 11g). Furthermore, resin extracted from dried inflorescences of HLVd-infected plants of genotypes ‘H6’ and ‘H10’ subjected to several processes commonly used to extract total cannabinoids and terpenes to produce resinous oils was assayed for the presence of HLVd. The viroid was found to be present in samples following CO_2_ extraction to produce “crude oil” and in samples of winterized oil with terpenes added back to produce “full spectrum oil”. It was not, however, detected in samples following CO_2_ extraction plus ethanol extraction followed by roto-evaporation to yield “winterized oil” (Figure 11h,i).

### 2.10. Transmission of HLVd Through Seeds and Pollen

#### 2.10.1. Hemp

**Figure 12:**
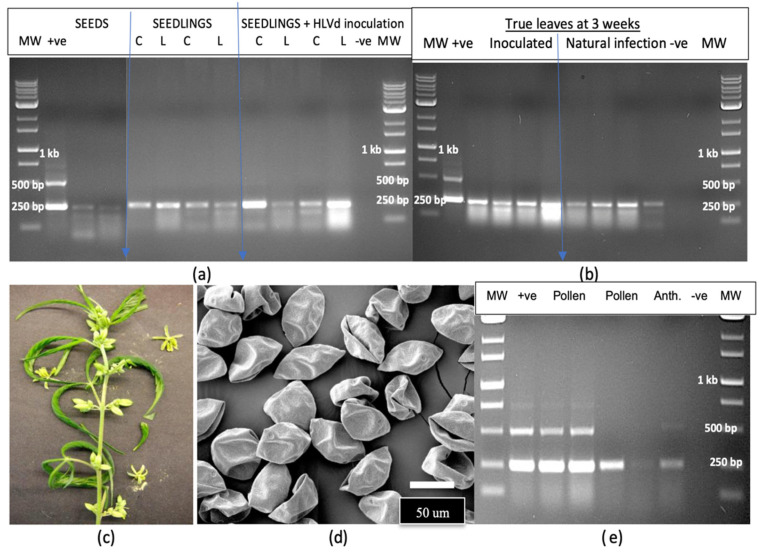
Detection of hop latent viroid in hemp seeds, seedlings and pollen grains. (**a**) The viroid was detected in seeds, cotyledons (C) and leaves (L) of seedlings from natural infection, and in cotyledons and leaves of seedlings following artificial inoculation (+HLVd). (**b**) Detection of HLVd in true leaves from artificially inoculated and naturally infected seeds. (**c**) A hemp plant producing male flowers and pollen. (**d**) Pollen grains from anthers following air-drying. (**e**) RT-PCR analysis shows the presence of HLVd in pollen and in anthers of hemp.

A sample of hemp seeds from an experimental breeding trial was tested for the presence of HLVd and a high proportion (60%) was found to be positive (Figure 12a). Seedlings emerging from these seeds, as well as from seeds that were artificially inoculated by immersing them in infectious sap prior to planting, produced seedlings that were positive for HLVd in the cotyledons and first true leaves (Figure 12a). The viroid was also detected in leaf samples collected after 3 weeks of growth from both sets of seeds (Figure 12b). Adult plants that naturally produced male flowers after 6 weeks of growth (Figure 12c) were used as a source of anthers and pollen for scanning electron microscopy and for RT-PCR analysis. Large amounts of pollen grains were produced, which appeared collapsed due to the air-drying used in the sample preparation (Figure 12d). RT-PCR analysis revealed the presence of HLVd in both the pollen and anthers from these hemp plants (Figure 12e), with the pollen samples displaying the characteristic multiple banding patterns for HLVd. The plants from which these samples were obtained were naturally infected with HLVd through seed-borne infection and developed no symptoms.

#### 2.10.2. Cannabis

**Figure 13:**
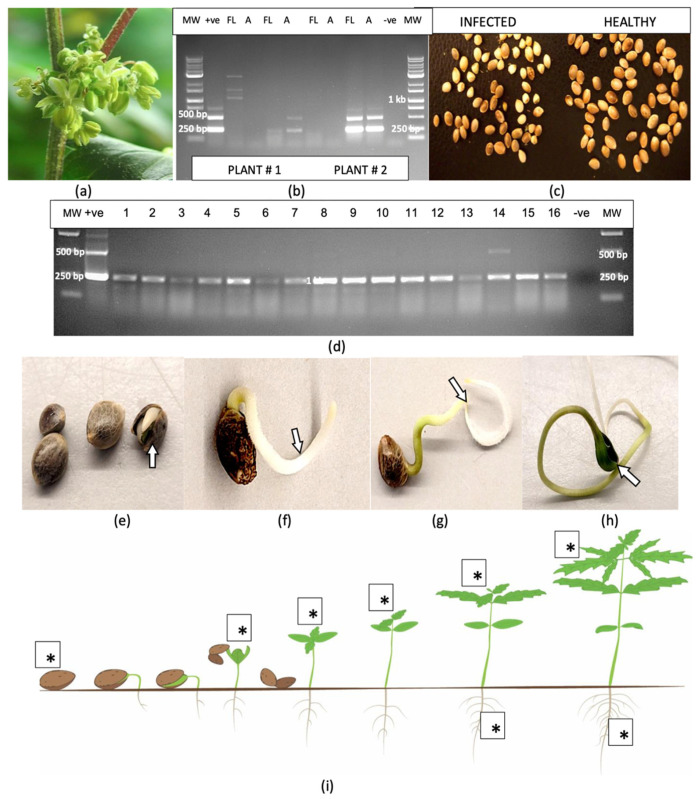
Detection of hop latent viroid in cannabis flowers, pollen, seeds and seedlings (**a**) Male flowers on a cannabis plant. (**b**) RT-PCR of whole flowers (FL) and anthers (A) from two cannabis plants showing presence of HLVd. (**c**) A comparison of seeds from an infected (left) and a healthy cannabis plant (right) showing the impact of HLVd on seed size and development. (**d**) Detection of HLVd in naturally infected seeds of cannabis showing the presence of a 256 bp band in all 16 seeds. (**e**) HLVd was detected on the seed coat of cannabis seeds. (**f**,**g**) HLVd was not detected in the emerging radicles from infected seeds (arrows). (**h**) HLVd was detected in the cotyledons (arrow). (**i**) Summary of seed germination and seedling emergence from infected cannabis seeds. Asterisks (*) denote where HLVd was detected.

The application of laboratory-grade silver nitrate under experimental conditions to induce male flowers led to clusters developing at the nodes (Figure 13a). An RT-PCR analysis of duplicate samples of flowers and anthers demonstrated the presence of HLVd in both samples (Figure 13b), with one plant showing a higher intensity of bands than the other. From the ‘M1’ × ‘G11’ cross, a large number of seeds were collected at maturity. A comparison of the morphology of seeds from HLVd-positive plants to those from HLVd-negative plants showed the former tended to be smaller and less fully developed compared to the latter (Figure 13c). A random sample of 16 seeds was tested for HLVd presence using RT-PCR. All seeds were positive for HLVd (Figure 13d), with a 256 bp band observed in all samples at different intensities; a weak band at 512 bp was only observed in one sample. The infected seeds were then germinated on moist paper towels and emerging roots and the primary root and cotyledonary tissues were sampled at various times during the 5–12 day period following radicle emergence and tested for HLVd (Figure 13e–h). The viroid was detected on the seed coat but not in the emerging radicle and was initially only detected in the fully expanded cotyledons and all true leaves that developed subsequently to that (Figure 13i). HLVd was absent in young root tissues until the first true leaves had fully expanded and was only detected in root samples collected after that (Figure 13i).

### 2.11. Survival of Hop Latent Viroid in Plant Tissues and the Surfaces of Utensils

#### 2.11.1. Plant Tissues

**Figure 14:**
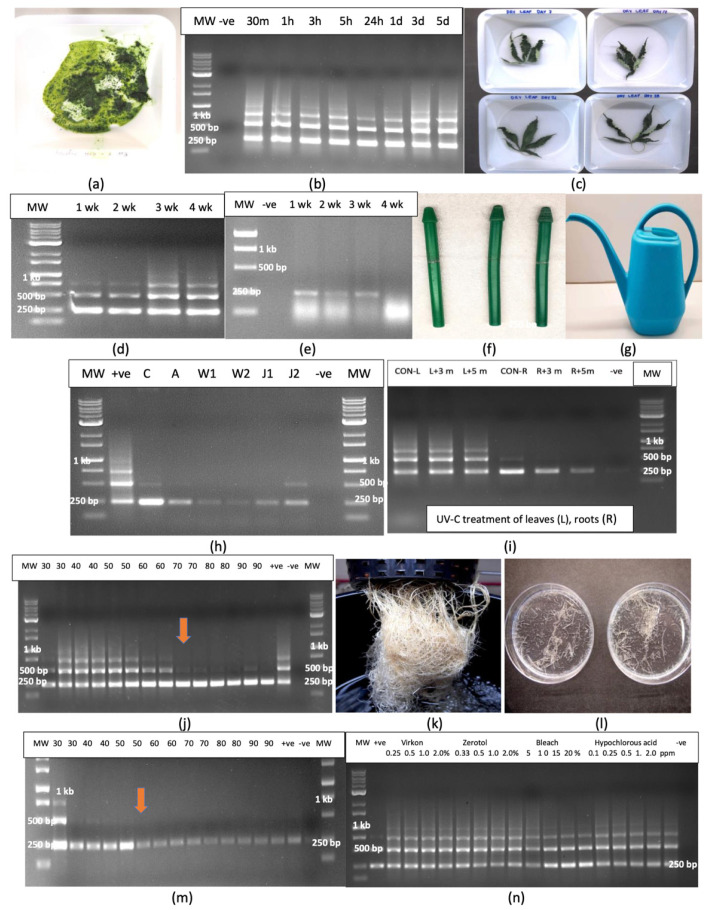
Survival of hop latent viroid in plant sap, in plant tissues and on the surfaces of utensils. (**a**) Dried plant sap placed in weighing boats was tested by RT-PCR at varying time periods. (**b**) The viroid was stable in sap for up to 5 days and displayed the characteristic multiple banding patterns at all time intervals. (**c**) Dried leaves from infected plants were left under laboratory conditions for periods of up to 4 weeks. (**d**) RT-PCR showed the viroid was detected in leaves for up to 4 weeks. (**e**) The viroid was detected in dried roots for up to 4 weeks. (**f**) Plastic disposable pestles used to grind plant tissues and (**g**) watering cans used to deliver nutrient solution were tested for the presence of HLVd. (**h**) RT-PCR analysis revealed the presence of a 256 bp band of HLVd in samples obtained from the surface of pestles (C), from the surface of autoclaved pestles (A), from nutrient solution in storage containers (W1, W2) and from the surface of watering cans (J1, J2). (**i**) Effect of UV-C irradiation on stability of HLVd in infected leaves and roots. Tissues were either not treated (CON) or exposed for 3 min or 5 min to a UV-C lamp. All samples showed the characteristic banding pattern of control samples. (**j**) Effect of heat on the stability of HLVd in plant tissues. Infected leaf tissues were exposed to treatments ranging from 30 °C to 90 °C for periods of 15 or 30 min. RT-PCR analysis showed that at temperatures up to 60 °C, multiple banding patterns were observed similar to the untreated control, while at 70 °C and higher, a single band of 256 bp was detected (arrow) and the higher MW bands were diminished in intensity. (**k**) Root samples from an infected plant grown in hydroponic containers were harvested, cut into small segments as shown in (**l**) and exposed to heat treatments. (**m**) RT-PCR analysis showed that at temperatures up to 50 °C, a single 256 bp band was observed similar to the untreated control, while at higher temperatures, the band intensity was diminished. (**n**) Exposure of intact leaves to four chemical treatments at varying concentrations for 2 min followed by RT-PCR showed that none of the treatments had an effect on the stability of the RNA of HLVd.

All samples of crushed leaf extracts (extracted sap) placed in weighing boats (Figure 14a) and tested after various time periods (30 min to 7 days) showed the characteristic multiple banding pattern for HLVd (Figure 14b). Similarly, intact leaves and roots that were left to dry for periods of 1 to 4 weeks (Figure 14c) yielded multiple bands characteristic for HLVd in leaves (Figure 14d); however, in root tissues, only a single 256 bp band was seen (Figure 14e).

#### 2.11.2. Utensils

Disposable plastic pestles used to grind plant samples that were autoclaved and reused (Figure 14f) and the surface of a plastic watering can that was used to deliver nutrient solution to cannabis plants (Figure 14g) all contained a faint 256 bp band that confirmed the presence of HLVd (Figure 14h).

#### 2.11.3. Effect of UV-C Treatment and Temperature on HLVd Survival

Samples of HLVd-infected leaves and roots were exposed to UV-C from a hand-held device for 3 min or 5 min. Leaves treated with UV-C did not affect HLVd stability and multiple banding patterns similar to that seen in the untreated control leaves were observed in treated leaves following RT-PCR (Figure 14i). In root tissues, a single 256 bp band corresponding to HLVd was observed in both control and UV-treated roots (Figure 14i); however, a 5 min exposure appeared to reduce the intensity of the band, suggesting partial degradation had occurred. The exposure of HLVd-infected leaves to temperatures ranging from 30 to 90 °C for 15 or 30 min showed no effect on HLVd stability and multiple bands were observed, similarly to untreated control leaves (Figure 14j). However, at higher temperatures (70–90 °C), the molecular weight bands corresponding to 512 and 768 bp were absent, suggesting they were possibly degraded at these temperatures (Figure 14j). Root samples from hydroponic culture (Figure 14k) were cut into small pieces (Figure 14l) and similarly exposed to temperatures ranging from 30 to 90 °C for 15 or 30 min. The results from the RT-PCR of treated samples showed no effect at 30–50 °C and a single 256 bp band was present in all samples (Figure 14m). At temperatures over 50 °C, however, the intensity of the band was visibly reduced, suggesting partial degradation occurred at these temperatures. HLVd was still detectable as a faint band in all samples, even at the higher temperatures.

### 2.12. Effect of Disinfectants and Chemicals on HLVd Integrity

#### 2.12.1. Whole Roots

The exposure of HLVd-infected root segments to treatments that included the commercial sanitation products Virkon (0.25–2%)and Zerotol (0.33–2%), as well as the laboratory sanitation products that included bleach containing 8.25% NaOCl (5–20%) and hypochlorous acid (0.1–2 ppm) for 2 min followed by rinsing, RNA extraction from treated and untreated tissues and RT-PCR showed that none of the treatments affected the stability of the viroid, and identical multiple banding patterns were observed in all samples. A similar lack of effect was seen in intact treated leaves (Figure 14n).

#### 2.12.2. Leaf Extracts

**Figure 15:**
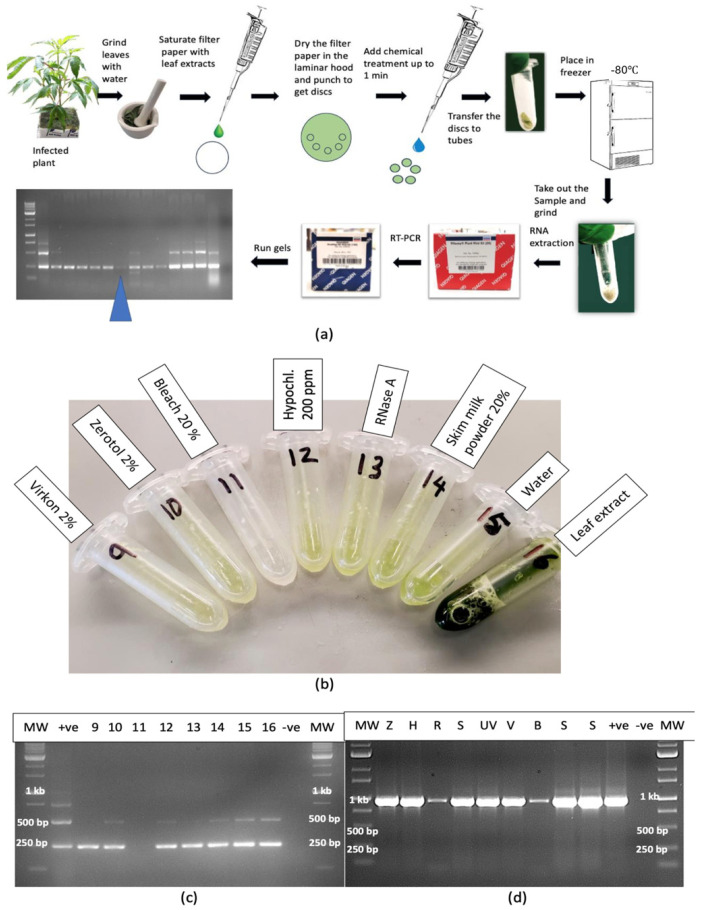
The effect of various treatments on the stability of HLVd in infected plant sap. (**a**) The method employed involved saturating filter paper discs with infectious sap and exposing air-dried discs to various chemical and enzymatic treatments for 1 min, following which the discs were transferred to Eppendorf tubes and stored in a −80 °C freezer until RT-PCR analysis could be conducted to determine if HLVd was detectable. (**b**) Eppendorf tubes containing filter paper discs were subjected to various treatments as indicated. Control discs received water. (**c**) RT-PCR analysis of RNA extracted from treated discs shows that only bleach at 20% destroyed the RNA to a point where it was not amplified. In the remaining treatments, a 256 bp band and a faint band at 512 bp could be seen similar to the control. (**d**) Exposure of RNA of *C. sativa* mitovirus (CasaMV1) to various treatments showed that RNase A (lane R) and bleach (lane B) reduced the intensity of the 980 bp band significantly compared to the other treatments which had no effect. The other treatments were 2% Zerotol (lane Z), 1000 ppm hypochlorous acid (lane H), untreated sap (lane S), exposure to UV-C light for 3 min (lane UV) and 2% Virkon (lane V).

A range of treatments applied to extracted sap from infected leaves added to dried filter paper discs for 1 min and transferred to Eppendorf tubes (Figure 15a,b) showed that only bleach at 20% degraded the RNA to a point where it was not amplified by RT-PCR (Figure 15c). The treatments that did not show an effect included Virkon (2%), Zerotol (2%), hypochlorous acid (200 ppm), RNase A (1 unit), skim milk powder (20%) and control (no treatment). For comparison, the same treatments were applied to extracted sap and the RNA was amplified with primers for CaMV1 (Figure 15d). The results showed that RNase A and bleach (20%) significantly reduced the intensity of the bands, suggesting that almost complete degradation had occurred (Figure 15d). The remaining treatments had no effect.

**Figure 16:**
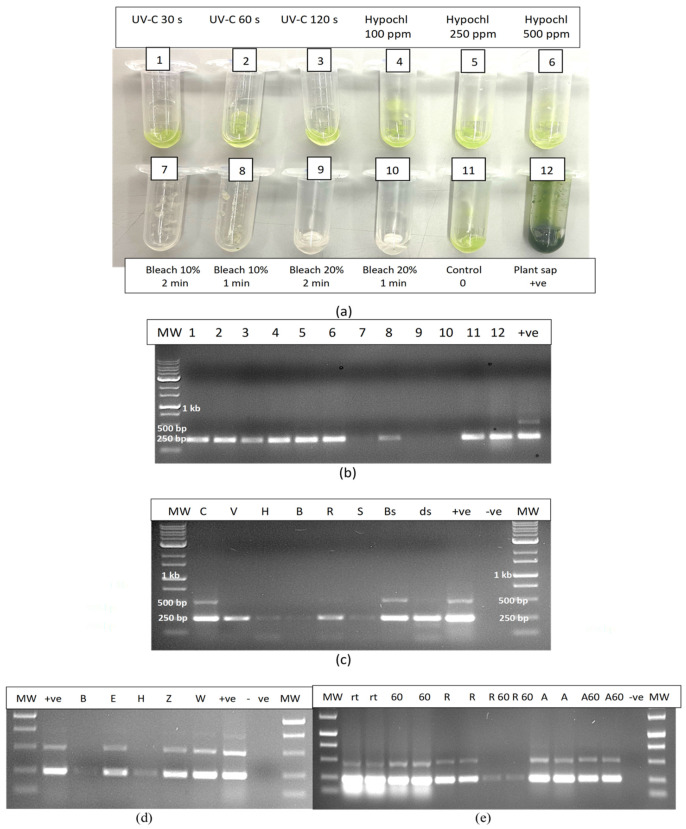
The effect of various treatments on the stability of HLVd in infectious plant sap. (**a**) Eppendorf tubes containing filter paper discs were subjected to various treatments as indicated. Control discs received water. (**b**) RT-PCR analysis of RNA extracted from treated discs containing sap shows that only bleach destroyed the RNA to where it was not amplified. The effective concentrations were 10% bleach for 2 min (lane 7) or 1 min (lane 8), 20% bleach for 2 min (lane 9) or 1 min (lane 10). In the remaining treatments, a 256 bp band was detected similar to the control. (**c**) RT-PCR analysis of RNA extracted from treated discs shows that the following treatments destroyed the RNA a point where it was not amplified: 1000 ppm hypochlorous acid (lane H), 10% bleach (lane B), and 20% skim milk powder (lane S). The other treatments in which the bands were still amplified included 2% Virkon (lane V), RNase A (lane R), culture extract from *Bacilllus subtilis* (lane Bs) and diluted sap (1:10). (**d**) RT-PCR analysis of RNA extracted from treated discs shows that the following treatments destroyed the RNA where it was not amplified: 10% bleach (lane B) and 1000 ppm hypochlorous acid (lane H). The other treatments where the RNA was not destroyed were 70% ethanol (lane E), 2% Zerotol (lane Z) and water (lane W). (**e**) RT-PCR analysis of RNA extracted from treated discs containing sap shows that the following treatments destroyed the RNA where it was weakly amplified: exposure to RNase A + 60 °C for 10 or 20 min (R60). The other treatments in which the bands were still amplified included room temperature (rt), exposure to 60 °C for 10 or 20 min (60), exposure to RNase A for 10 or 20 min (R), exposure to RNase A for 10 or 20 min (A) or exposure to RNase A + 60 °C for 10 or 20 min (A60).

**Figure 17:**
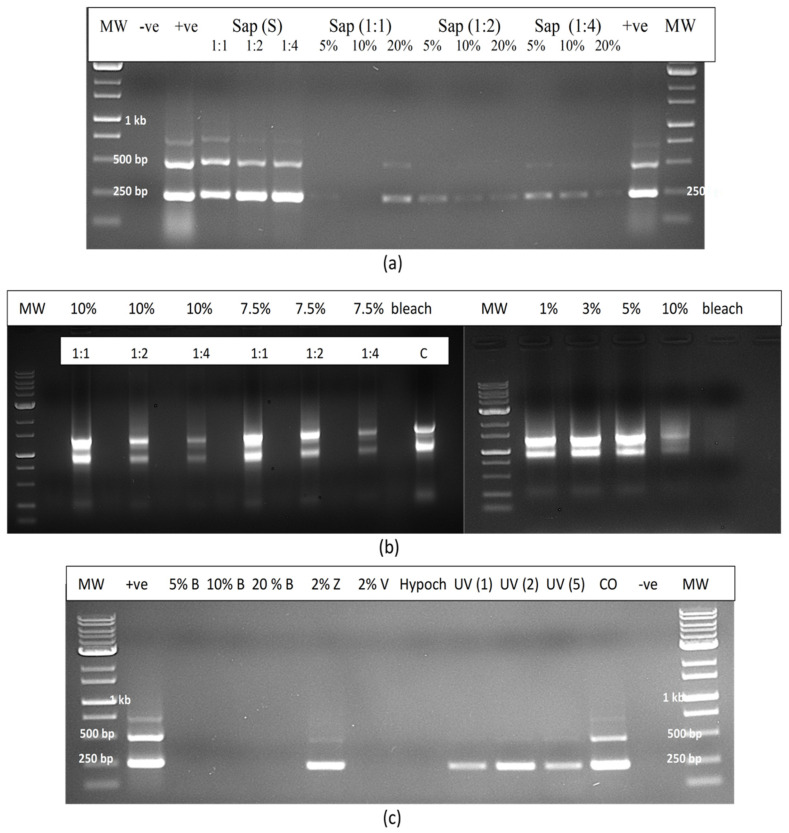
(**a**) RT-PCR analysis of RNA extracted from infectious sap shows that the following treatments destroyed the RNA since it was not amplified or was only weakly amplified: 5%, 10% and 20% bleach (8.25% NaOCl) with sap diluted by 1:1, 1:2 and 1:4. (**b**) The effect of varying concentrations of bleach on the degradation of total RNA extracted from plant tissues that includes 18S/16S RNA. In the left panel, total RNA was undiluted (1:1) or diluted 1:2 and 1:3 and then exposed to 10% or 7.5% bleach. The bleach degraded more RNA at the 1:3 dilution. In the right panel, undiluted total RNA was subjected to bleach concentrations ranging from 1% to 10%. The highest concentration (10%) caused the most degradation. (**c**) Water containing HLVd added as infectious sap was subjected to a range of treatments, following which RT-PCR was conducted to determine amplification. Treatments that did not produce a band were bleach (B) containing 8.25% NaOCl at 5, 10 and 20%, 2% Virkon in water (V) and 600 ppm hypochlorous acid. Positive controls included infectious sap (+ve) and sap added to water (CO), which showed multiple bands were amplified. In the UV treatments (1, 2 and 5 min), the higher-molecular-weight band (512 bp) representing linear RNA of HLVd was destroyed but the 256 bp band was unaffected.

The experimental treatments applied to extracted sap were repeated to include additional treatments such as UV-C exposure for 30, 60 and 120 s, hypochlorous acid at 100, 250 and 500 ppm and bleach at varying concentrations and exposure times (Figure 16a). The results showed that only bleach at 10% for 2 min or at 20% for 1 or 2 min completed degraded HLVd RNA, while 10% bleach for 1 min caused partial degradation as determined by RT-PCR (Figure 16b). Further treatments that completely degraded the RNA of HLVd in extracted plant sap were 1000 ppm hypochlorous acid for 1 min and skim milk powder (20%) for 20 min (Figure 16c,d). Treatments that had no effect included a 10 min exposure to a culture extract from the biocontrol agent *Bacillus subtilis* (Figure 16c) and to 70% ethanol for 10 min (Figure 16d). The addition of RNase A (1 unit) to extracted sap only partially degraded the RNA of HLVd (Figure 16c); however, when combined with exposure to 60 °C for 10 or 20 min, the RNase A degraded the RNA but the addition of RNase R had no effect (Figure 16e). In further experiments, dilutions of plant extract in water (1:1, 1:2 and 1:4) were tested with varying concentrations of bleach and hypochlorous acid. Bleach at 5, 10 and 20% was effective in degrading the RNA of HLVd at all dilutions (Figure 17a). The exposure of total plant RNA extracted from cannabis leaves to varying concentrations of bleach showed that, at 10%, the RNA was degraded, especially when the initial RNA concentration was diluted 1:4 (Figure 17b).

#### 2.12.3. Contaminated Water

In evaluating the efficacy of various treatments on the degradation of HLVd RNA added to water as infectious plant sap, bleach (5–20%) and hypochlorous acid (600 ppm) were shown to be effective once again, and no band was observed following RT-PCR compared to the untreated control, which showed multiple bands (Figure 17c). Treatment with UV-C for 1–5 min had no effect but Virkon at 2% degraded the RNA of HLVd whereas, in previous experiments, this chemical was ineffective when plant sap was treated compared to sap diluted in water.

### 2.13. Meristem Tip Culture

**Figure 18:**
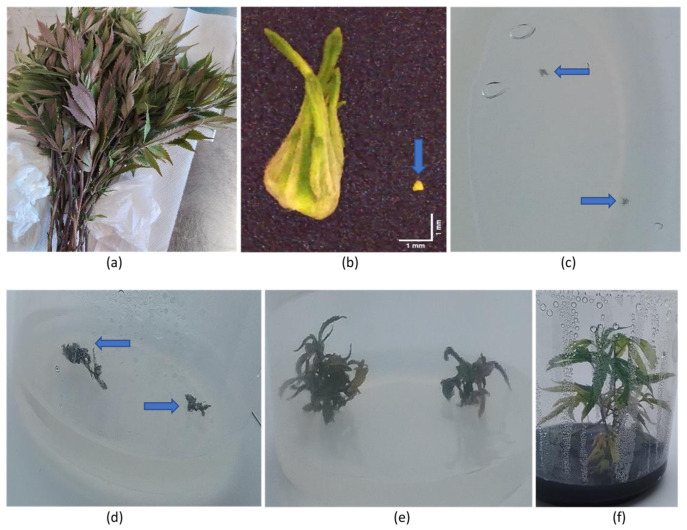
Meristem tip culture for recovery of HLVd-free explants. (**a**) Cuttings taken from the middle branches of a two-month-old HLVd-infected mother plant. (**b**) An excised shoot tip (left) and meristem excision of 0.4 mm size (bottom right, arrow) with two or less leaf primordia from the shoot tip. Scale bar = 1 mm. (**c**) First subculture (three-week growth) of meristematic explants (arrows) on tissue culture medium. (**d**) Second subculture (six-week growth) of meristem explants (arrows); the first HLVd test was conducted during this subculture. (**e**) Fourth subculture (12-week growth) of meristem explants. (**f**) Eighth subculture (24-week growth) of meristem explants. Results from testing for presence/absence of HLVd in these tissue-cultured plants are presented in Table 3.

Cuttings taken from HLVd-infected mother plants of eight genotypes (Figure 18a) were used as a source of shoot tips (Figure 18b) from which meristems measuring around 0.4 mm in size were dissected (Figure 18b). The meristems were transferred to tissue culture medium, where after 3 weeks, they were barely visible on the agar medium (Figure 18c). Shoot development was observed 6 weeks after the meristems were placed on tissue culture medium (Figure 18d), followed by more extensive shoot growth at 12 weeks (Figure 18e) and after 24 weeks (Figure 18f). The developing shoots were tested for the presence of HLVd every six weeks for a period of 6 months using RT-qPCR. The data from the 6-month sampling date are presented in Table 3. A total of 91 meristems were recovered from the eight genotypes, and the average frequency of HLVd-negative plants was 40.66%. However, there were significant differences among the eight genotypes in the frequency of pathogen-free shoots, which ranged from nil to 100% (Table 3). Among the genotypes that had HLVd-positive shoots, the viroid levels were high, indicating replication was occurring within the in vitro-grown shoot tissues.

### 2.14. Summary of Hop Latent Viroid Spread

**Figure 19:**
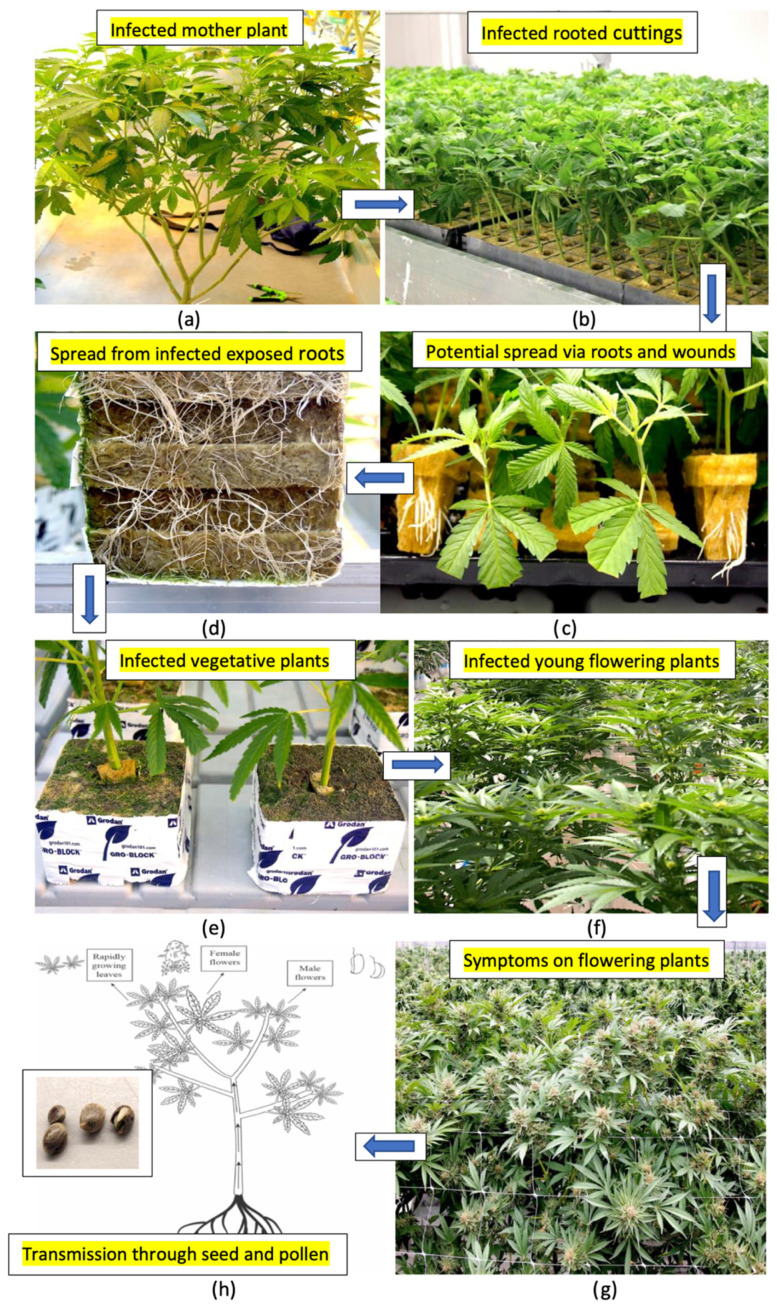
Summary of the various avenues through which HLVd can be spread in an indoor hydroponic cannabis-growing environment based on the results from this study. Starting from infected stock plants (**a**), most of which are asymptomatic, cuttings rooted in a propagation room were shown to be infected at a high frequency and mostly remained asymptomatic (**b**,**c**). These rooted cuttings gave rise to infected vegetative plants (**d**), which may show symptoms of stunted growth and reduced root development. Spread of HLVd from exposed roots and through wounds can take place during the vegetative plant stage (**e**), especially through recirculated nutrient solution. Infected flowering plants originating from infections that occurred at the vegetative or propagative stage can display symptoms of stunting and reduced growth when placed under a 12:12 h photoperiod to induce flowering (**f**). These plants continue to display symptoms throughout the flowering period, including smaller inflorescences and reduced cannabinoid production (**g**). Plants grown for seed that are infected can produce infected pollen and seed at a high frequency, resulting in further spread of the viroid (**h**).

Based on the results from this study, the various methods by which HLVd can spread in a cannabis greenhouse cultivation environment are summarized in Figure 19. From infected stock plants (Figure 19a), cuttings were shown to be infected at a high frequency (Figure 19b,c) that also gave rise to infected vegetative plants at a high frequency (Figure 19d). The extent to which spread could be occurring within the propagation room between cuttings was not determined. Spread of the viroid from exposed roots of vegetative plants (Figure 19d) and through the wounding of roots can also take place during the vegetative plant stage (Figure 19e). The viroid can also be present on the surfaces of tables and in recycled hydroponic nutrient solution to result in infections at this stage. When these infected vegetative plants are transferred into the flowering rooms, infected plants originating from these infections previously taking place at the vegetative or propagative stages can display symptoms of stunting and reduced growth under the 12:12 h photoperiod conditions used to induce flowering. The extent of viroid spread from one infected flowering plant to adjacent plants was not determined. Plants grown for seed that are infected can produce infected pollen and seed at a high frequency, resulting in the further spread of the viroid.

## 3. Discussion

The findings from this study have identified four major avenues by which HLVd can be spread between cannabis plants in a greenhouse cultivation environment. First and foremost was the high frequency of transmission through cuttings that were derived from infected stock plants. These cuttings, once they had rooted, showed viroid presence at rates of up to 100% within 4 weeks of vegetative propagation, particularly when highly susceptible genotypes of stock plants were systemically infected with HLVd. Hence, frequent testing of stock plants and vegetative clones derived from them, followed by the eradication of diseased plant materials, is recommended for preventing the spread of HLVd within a greenhouse cultivation environment. This approach has been shown to be effective in reducing the spread of this pathogen [4]. This is consistent with eradication measures aimed at reducing the spread of other viroids, where destruction of infected plant materials was shown to reduce the incidence of important viroids, including potato spindle tuber viroid (PSTVd) [28,31]. In cannabis, eradication is particularly important since vegetative propagation from cuttings remains the most common method to initiate new cycles of plant production [1]. Where available, a disease certification programme that provides viroid-free plants would be the best strategy to reduce the spread of HLVd, but such a programme does not currently exist.

The distribution of HLVd in the foliage of systemically infected stock plants can be non-uniform; hence, not all tissue samples collected for testing will yield positive RT-PCR results [2]. The sampling of roots has been shown to be a more reliable method for detection, since root tissues are more consistently and uniformly infected by HLVd [2,16]. The results from the present study demonstrated that roots tended to accumulate HLVd before it was detected in the foliage, regardless of the inoculation method used. This is consistent with the mode of spread of a range of plant viruses, which frequently can be detected in the roots before spread occurs to the foliar tissues [39]. In a previous study [16], it was reported that HLVd-infected cuttings rooted to a much lesser extent when compared to healthy cuttings, consequently resulting in plants with a weaker root system. Similar observations were made on four cannabis genotypes in the present study, suggesting a negative impact on rooting frequency.

The rate of spread of HLVd from infected cuttings into developing roots during vegetative propagation may be influenced by the cannabis genotype being grown, as well as by environmental conditions such as temperature and light. For example, the shading of tomato plants was shown to delay the movement of PSTVd from the site of inoculation but enhanced downward movement into the roots [40]. A delayed movement of the viroid into root tissues was observed in one cannabis genotype in this study, but eventually cuttings of all genotypes succumbed to HLVd infection. In *Argyranthemum* plants infected with chrysanthemum stunt viroid, the rate of movement of the viroid into shoot apical meristems was also influenced by the genotype [41]. An analysis of flowering plants of one cannabis genotype naturally infected with HLVd in the present study showed that while all symptomatic plants contained the 256 bp band, confirming the presence of HLVd, the banding patterns and intensity were variable between adjacent plants—some plants displayed the characteristic multiple bands while others contained a single band. This suggests that different rates of replication of the viroid may be occurring within individual plants of the same genotype, even when grown adjacent to one another under the same environmental conditions. This plant-to-plant variation in the uptake of the viroid may complicate inoculation experiments aimed at demonstrating viroid transmission.

A second efficient method for the transmission of HLVd is through infected seed. These seeds may be derived from either an infected male or female plant serving as parent plants. Female inflorescences of cannabis were previously shown to contain high levels (genome copies) of the viroid [2], suggesting that spread into the ovules can occur readily to give rise to high rates of seed infection. The presence of HLVd in the anthers and pollen of male cannabis and hemp plants was confirmed in this study. This finding was unexpected given previous reports of a low frequency of HLVd presence in the pollen of hop plants (around 6%) [42], which was reported to be due to the activity of RNases that degraded the RNA of the viroid [43]. In contrast, a number of Pospiviroids, including PSTVd, were demonstrated to be efficiently transmitted through the ovules and pollen [25,28,44,45], similar to the rates reported for HLVd in the present study. In seeds derived from a cross made between cannabis genotypes ‘M1’ (female) and ‘G11’ (male), up to 100% were HLVd-positive. It was later confirmed that both parents harboured HLVd (‘M1’ was earlier found to be negative but became positive for HLVd prior to flowering). In instances where only one parent is infected with HLVd, the transmission rate is likely lower since only one gamete contains the viroid. Infected ovules likely give rise to a higher proportion of infected seeds when compared to infected pollen as a source of HLVd, since high copy numbers of the viroid within the inflorescence tissues of infected female plants were previously reported [2]. The results from the present study show, however, that pollen grains produced in both cannabis and hemp male flowers can harbour HLVd.

Cannabis or hemp genotypes appear to differ in their susceptibility to HLVd (defined as the effect of genotype on the relative rates of viroid replication and spread), giving rise to different copy numbers of the viroid in the plant. In inflorescence tissues, HLVd levels (as quantified by ddPCR) were shown to be significantly influenced by the genotype, with some highly susceptible genotypes accumulating much higher levels of the viroid [2]. This can influence the transmission rates of the viroid through seed but more research is needed to establish this genotypic effect. HLVd may be present both on the seed coat and in the embryo of infected cannabis seeds (Tassa Saldi, Tumi Genomics, personal communication), and these seeds can give rise to infected seedlings at a high frequency. The inoculation of seeds by dipping them in infectious sap gave rise to infected seedlings in the present study. Interestingly, the viroid was detected in the young developing leaves before it was detected in the root tissues. In general, foliar tissues appeared to support greater viroid replication than root tissues, based on RT-qPCR measurements made on stock plants. The role of seed-borne inoculum in spread of HLVd on cannabis or hemp plants grown under field conditions and the impact on subsequent seedling growth and inflorescence development is unknown and warrants further investigation.

A third method of transmission, reported for the first time in this study, was the potential for the spread of HLVd from infected roots. Infected plants grown in close proximity to healthy plants under hydroponic cultivation acquired the viroid in several experiments designed to demonstrate this mode of transmission. The source of inoculum is likely to originate from root fragments and sloughed-off root cells that contain the viroid, since there is no evidence that HLVd can persist outside of a host cell in the environment as infectious entities. In a trial greenhouse environment, HLVd was subsequently detected in recirculating nutrient solution, in run-off nutrient solution, on the surfaces of tables, and in various other locations. In an indoor growing room, the viroid was detected in nutrient storage tanks and on the surface of plastic jugs, although transmission from these sources was not demonstrated. The exposed root system in a hydroponic cultivation system makes the root-to-root spread of HLVd likely, and clusters of infected plants were observed on propagation tables, suggesting a focal point of inoculum, likely from an infected plant (authors, unpublished observations). There are previous studies which have demonstrated that plant viruses and viroids can be spread in recirculating nutrient solution in indoor growing environments, as well as through infections occurring via the root system, such as on pepper, potato and tomato plants [46,47,48]. Mehle et al. [46] reported that PSTVd could remain infectious in water for periods of up to 7 weeks at 20 ± 4 °C. In the present study, artificial inoculations conducted on cannabis and tomato plants in the indoor growing room demonstrated a more rapid uptake of the viroid into the plant from inoculated roots when compared to cut-stem inoculations under identical growing conditions. Therefore, the spread of HLVd through root infections is likely to occur under hydroponic cultivation conditions. The extent to which this mode of transmission also occurs in a soil-based production system, where the root zone is buffered by surrounding soil and associated microflora, is unknown but is likely to be lower. As such, the production of cannabis in soil cultivation may lead to lower infection levels resulting from root infections by HLVd compared to hydroponic cultivation.

A fourth mode of transmission of HLVd is through wounds created on stems through pruning activities or following the removal of cuttings for propagation. The placement of infectious sap directly on these wounded surfaces was demonstrated to lead to the transmission of HLVd. Such transmission could occur from contaminated pruning tools spreading sap from an infected plant to a healthy plant. The potential for a similar mechanical transmission taking place through wounded leaves in the greenhouse production environment has not been demonstrated. Our observations suggested that extensive wounding, i.e., the tearing of leaves, was required for HLVd transmission to occur. Therefore, unlike PSTVd, which can be spread through abrasions created by leaves rubbing against each other or through workers handling plants (35), such a mode of transmission has not been demonstrated for HLVd under commercial growing conditions. The patterns of distribution of diseased plants in a greenhouse suggest that infected cuttings are the primary inoculum source, followed by spread of the pathogen from infected roots or through recirculated contaminated water. Plant-to-plant spread through the touching or handling of plants appears to be relatively uncommon. Similarly, the transmission of HLVd by insects such as thrips, aphids and leafhoppers acting as vectors is likely to be low under greenhouse conditions, as their populations tend to be well managed. In contrast, insect transmission of HLVd is probably of greater importance outdoors in large-scale hemp production fields due to the greater abundance of these insects, which can result in the transmission of multiple viruses/viroids simultaneously [49].

The meristems of many plant species are tissues in which viroid presence and replication are reported to be considerably lower compared to differentiated leaf and stem tissues; as such, meristem tip culture has been demonstrated to result in the recovery of virus and viroid-free plants [50]. This approach was shown in previous studies to be successful in deriving HLVd-free hop plants [51,52,53]. In the present study, very small meristematic tissues (measuring 0.4 mm in size) were cultured on tissue culture medium and gave rise to plantlets that were shown to be free of the viroid. The frequency of HLVd-negative plants was significantly influenced by the plant genotype and ranged from nil to 100%. The plants continued to test negative for a period of up to 6 months, suggesting the viroid was absent at least up to that time. However, more testing over a prolonged period is needed to confirm the viroid is totally eliminated.

Meristem tip culture can potentially be implemented to establish viroid-free stock plants that can subsequently be used to start new plantings of cannabis. However, since the success rate was genotype-dependent, some genotypes may yield a low to no frequency of viroid-free plants. In such genotypes, the viroid levels were observed to increase during the tissue culture phase and accumulated to high levels in the leaves. In hop plants, meristem tips measuring <0.5 mm in size obtained from plants exposed to a cold treatment at 4 °C for one month yielded a high frequency of HLVd-negative plants, which was also dependent on the cultivar of hops used [51,52]. The influence of a cold treatment on the recovery of HLVd-negative plants of cannabis has not been investigated. The basis for the differential success rate in the meristem tip culture of different genotypes of cannabis or hops is also not known. In *Argyranthemum* plants infected with chrysanthemum stunt viroid, the rate of movement of the viroid into the shoot apical meristems was influenced by plant genotype [41]. It was demonstrated that in the more tolerant genotypes, callose deposition (containing beta-1,3 glucans) within the plasmodesmata was higher, potentially restricting the cell-to-cell movement of the viroid. Similar callose depositions may occur within cells of the cannabis cultivars in which meristem tip culture successfully eliminated HLVd but more research is required to confirm this. It is also not known if callose deposition within plasmodesmata could explain the differential response of cannabis genotypes to HLVd infection reported in a previous study [16].

The environmental and host factors that influence the extent to which HLVd replicates and spreads within cannabis plants have not been determined, due to a paucity of prior research on this recently reported pathogen. As a member of the family *Pospiviroidae*, however, HLVd is known to replicate in the plant nucleus and relies completely on its host for the replication cycle to be completed. Transport out of the nucleus involves a complex of transport proteins [54]. The asymmetric rolling circle mechanism that produces new linear viroid copies was confirmed in the present study, since the circular and linear forms of the viroid could be distinguished from each other by the different-sized banding patterns observed following RT-PCR, and by their different susceptibilities to degradation by RNases A and R. The multiple banding patterns, where present, likely represent an actively replicating viroid (high replication phase) when compared to a single 256 bp band commonly observed in seeds and in roots, suggesting lower replication levels (low replication phase) in these tissues. The factors that cause a shift from low to high replication phases are not known. In the present study, the exposure of infectious sap to temperatures over 60 °C resulted in the degradation of the higher-molecular-weight bands, suggesting they are more sensitive to denaturation at these temperatures, likely because they were linear concatamers. The sampling of a number of infected flowering plants of the same genotype in a trial greenhouse study revealed different intensities of banding patterns, possibly reflecting the differing rates of replication and viroid accumulation among individual plants derived from the same stock plants, the basis for which remains unknown. The secondary structures of the HLVd and PSTVd rod-shaped molecules may influence their infectivity. In PSTVd, the RNA secondary structure was shown to be important for infectivity [55]. Nucleotide deletions or substitutions that affected the secondary structure in PSTVd also influenced viroid movement (trafficking) through the phloem [23,56].

The detection of HLVd in root tissues within 2–3 weeks following stem inoculation, followed by sequential detection in the foliage by 4 weeks, usually in the uppermost part of the plant, is consistent with the movement pattern of many plant viruses. This pattern of spread occurs by cell-to-cell movement via the plasmodesmata and transfer into the phloem tissues, within which viruses and viroids are transported [21,23,25,40,41,56,57,58]. Actively growing tissues, such as shoot and root tips, are active sites of virus (and presumably viroid) accumulation [57]. The rate of spread of HLVd through the phloem of cannabis plants into the floral tissues was enhanced in the present study by placing inoculated plants under a 12:12 h photoperiod, which induces rapid and dramatic changes in the morphology and physiology of the plant as it prepares to initiate flowering [58,59]. The impact of the light duration, per se, was ruled out by exposing autoflower genotypes, which flower regardless of light duration, to the 12:12 h photoperiod [60]. Viroid accumulation was not directly affected by this photoperiod change but rather by the initiation of the flowering process, which resulted in the rapid distribution of the viroid in all tissues of the autoflower plants during flowering, regardless of the photoperiod. The increased demand for photosynthates required by the developing inflorescences that contain numerous glandular trichomes acting as a metabolic sink [17,61] would simultaneously have caused the rapid movement of the viroid into the developing floral tissues, where it subsequently negatively impacts trichome development and reduces cannabinoid accumulation [16]. Tabler and Tsagris [23] indicate that systemic spread is not passive but requires specific RNA motifs and host factors. At the early stages of inflorescence development in infected cannabis plants, the symptoms due to HLVd become readily apparent and steadily worsen [16], likely as a result of enhanced viroid replication and spread. In Arabiopsis plants artificially inoculated with two RNA viruses (a Potyvirus and a Tobamovirus), the transition to flowering resulted in an increase in virus levels in the aerial plant parts, accompanied by a significant decline in the levels found in the roots [62]. Whether a similar inverse correlation occurs in cannabis plants has not been investigated.

HLVd was shown to be capable of invading the trichomes in cannabis inflorescences and was present in a number of dried samples prepared and tested during the trial. Several plant viruses have been reported to enter into plant trichomes [63,64,65], the biological significance of which is not yet known. We provide the first evidence of the presence of HLVd within cannabis trichomes. The unexpected amplification of HLVd from resinous materials extracted from cannabis trichomes using several methods suggests it is a remarkedly stable viroid. This was confirmed by experiments aimed at establishing the effect of temperature extremes on viroid stability. The exposure of infected leaves and roots to temperatures of 60–80 °C for 15–30 min did not affect the integrity of the viroid, and the amplification of HLVd from the surface of reused plastic pestles that were autoclaved at 120 °C for 15 min support its tolerance to extreme heat conditions. The complementary base-pairing and stem loop structures depicted from the RNA sequence using rna-fold likely contributed to its tolerance to environmental extremes. The covalently closed form of circular RNA’s generally provides them with a longer half-life and more resistance to RNase R than linear RNA’s [66,67]. The exposure of infected leaf and root tissues to UV-C irradiation for up to 5 min similarly did not destroy the circular RNA of HLVd. However, the linear forms of higher molecular mass showed greater degradation at temperatures exceeding 60 °C and after UV-C exposure in this study, as expected. Since HLVd replication occurs within the nucleus, the stable circular viroid form and intermediate linear forms would also be localized within the nucleus and/or nucleolus [20,21,25,54]. After these attempts to denature the RNA of HLVd in leaf extracts, follow-up infectivity experiments could not be conducted since HLVd does not produce noticeable symptoms in cannabis plants until they begin to flower, except for highly susceptible genotypes that show some stunting during vegetative growth.

A method was developed in the present study to screen plant sap containing HLVd RNA for sensitivity to degradation by chemicals, which allowed a range of products to be tested in vitro. After these treatments, the RNA of HLVd was shown to be consistently degraded by exposure to ribonucleases, bleach and hypochlorous acid. Bleach containing NaOCl is known to denature nucleic acids [68], making it the chemical of choice for disinfecting greenhouse surfaces and tools to eliminate viruses and viroids, in addition to other plant pathogens [15,30,32,33,34,68]. Sodium hypochlorite “destroys” DNA through oxidative damage, such as base modifications and the production of chlorinated base products. The exposure of DNA to increasingly higher concentrations of NaOCl causes cleavage of the strands, breaking the DNA into smaller and smaller pieces, and eventually into individual bases [69]. Prince and Andrus [70] determined that 10% (*v*/*v*) Clorox bleach (equivalent to ~0.55% sodium hypochlorite) was effective in destroying DNA, while 2.5% (*v*/*v*) Clorox bleach only caused nicking of the DNA, as evidenced by the slower mobility of the treated DNA during gel electrophoresis. In the present study, bleach products containing 7.55–8.25% NaOCl were shown to be effective at levels ranging from 5% to 20%. Since bleach products may contain different levels of NaOCl, ranging from 5.5% (common household bleach) to 8.25% (industrial bleach), achieving minimal levels comparable to that provided by 5% of 7.55% NaOCl is required. Hypochlorous acid (HOCl) is formed when sodium hypochlorite is added to water at a pH 5.5–7.0, thus acting in the same manner as NaOCl. Other chemical products, including Virkon, while not affecting viroid RNA integrity in vitro, may still be recommended as a cleaning agent to reduce virus and viroid transmission [32,67]. When added to water containing HLVd, Virkon at 2% reduced viroid integrity but had no effect when it was added directly to plant sap. Skim milk powder partially degraded the RNA of HLVd in vitro and has been shown to reduce the transmission of PSTVd in other studies [30,32,33]. It is assumed that the enzymes present in skim milk negatively affected RNA stability. The addition of skim milk to infectious sap directly, however, did not fully reduce the integrity of the RNA. Ethanol had no effect on HLVd, confirming results from other studies regarding its inefficacy in reducing the survival of viroids affecting plants [33,67]. The efficacy of the various treatments tested in degrading HLVd RNA was affected by initial viroid levels, since diluted sap showed a greater sensitivity to the chemicals compared to sap containing higher levels of viroid RNA. The addition of RNase A to infectious sap did not destroy the RNA when compared to in vitro studies involving purified RNA, perhaps due to the presence of inhibitors in the plant sap. However, high-temperature (60 °C) treatment coupled with the addition of RNase A rendered the viroid RNA more susceptible to degradation. This suggests that the combined treatment degraded the RNA of HLVd in sap.

Artificial inoculation experiments conducted with tobacco and tomato plants revealed that both were capable of supporting the replication of HLVd, but the spread of the viroid following cut-stem or petiole inoculations took considerably longer (4–8 weeks) when compared to inoculations performed on cannabis plants (2–3 weeks). Only a single 256 bp band was observed in all tissue samples tested, suggesting that the viroid was in the “low-replication phase” in these plants, as multiple banding patterns indicative of high replication activity, commonly seen in cannabis plants, were not observed. The practical significance of this expanded host range for HLVd is unclear at the present time, since it has not been demonstrated that these plants could act as alternate natural hosts or reservoirs for HLVd and further studies are needed in this regard. However, these two plant species can be used as alternate hosts in which to study HLVd dynamics in a susceptible host other than cannabis. The host range of many plant viroids is known to be wide. For example, PSTVd can infect over 100 plant species and the hop stunt viroid infects plants in several different families [24,25,26,27]. Additional plant species are likely to be identified as potential hosts following artificial inoculation studies with HLVd. A previous study demonstrated that both tomato and *Nicotiana benthamiana* plants supported the replication of an isolate of HLVd from hops that underwent heat treatment, resulting in mutations in the viroid [71]. These mutations resulted from sequence changes at various positions in the secondary structure [72].

The changes in gene expression levels and transcriptomic studies in the HLVd–cannabis interaction warrant further research. These studies would provide a better understanding of the mechanisms by which the various symptoms develop following HLVd infection, as well as potentially identifying mechanisms by which viroid replication can be reduced. The characterization of cannabis genotypes with tolerance or resistance to HLVd remains a high priority, and knowledge of the underlying mechanisms and changes in gene expression would greatly aid in managing this pathogen. The appearance of new strains of HLVd with altered virulence through adaptive mutations is an ongoing concern as these are commonly reported to occur in viroids, resulting in the appearance of “quasi-species” [24]. Currently, the genetic diversity in HLVd appears to be limited [2] but continuous monitoring for the development of new genetic variants should remain a priority. These variants may have altered virulence patterns or host ranges. Suzuki et al. [73] demonstrated that apple fruit crinkle viroid, a member of the Pospiviroidae, when inoculated onto experimental hosts such as tomato, cucumber and wild hops, underwent sequence changes affecting 2–10 nucleotides from the original isolate. Similarly, a single nucleotide substitution converted PSTVd from a non-infectious to an infectious RNA on *Nicotiana tabacum* [74]. These studies point to the potential for additional variants of HLVd to develop over time, which would continue to pose ongoing threats to the cannabis industry in North America. The results from this study provide information on numerous aspects of the epidemiology of HLVd and identify several management options to reduce the economic impact of this viroid.

## 4. Materials and Methods

### 4.1. Plant Materials and Growing Conditions

This research was conducted in collaboration with several Health Canada-approved licenced facilities in which different genotypes (strains) of high Δ^9^-tetrahydrocannabinol (THC)-containing cannabis (*Cannabis sativa* L.) plants were cultivated under greenhouse conditions using hydroponic methods of production [1]. In addition, plants were also grown within an indoor facility with supplemental lighting under hydroponic conditions described by Scott and Punja [13] to perform controlled studies on viroid transmission, spread and longevity. Several different cannabis genotypes were included in this study, representing those most commonly grown during the period over which this research was conducted (2021–2023). Many of these genotypes were susceptible to HLVd, as determined by characteristic symptoms that developed following natural infection [16]. All plants originated from vegetative cuttings taken from stock (mother) plants and were maintained in a dedicated greenhouse nursery area with a temperature range of 23–28 °C and relative humidity of 60–70%. The stock plants were grown in the nursery area for periods of 3–6 months and were sampled at various times to confirm the presence of HLVd using a molecular assay involving RT-PCR (see below).

For the propagation of cannabis plants for extensive production, cuttings (clones) originating from stock plants were dipped in a rooting powder (containing indoleacetic acid) and inserted into 2.5 cm^3^ rockwool cubes and placed in trays in a dedicated propagation room under high relative humidity (80–90%) and a temperature range of 23–27 °C to induce root development. After 2 weeks, rooted cuttings were transferred into wells cut into 10 cm^3^ rockwool blocks and placed on a greenhouse bench for an additional 2–3 weeks to allow vegetative growth to continue. During this time, the shoot tip on the leading stem of each plant was manually removed (pinching) to promote lateral growth. A select number of these vegetative plants were set aside in the present study and designated as future stock plants. The remaining plants were placed onto large cocofibre blocks, one plant per block, and transferred to a large flowering room. After one week, the photoperiod was adjusted to 12 h light–12 h darkness to induce flowering [1]. The water and nutrient regimes were provided according to recommended growing requirements to ensure optimal growth. The plants were trained and supported by plastic netting that ensured the developing inflorescences remained upright. The pruning of leaves and training of plants were conducted manually according to growing requirements as needed. Plants were grown for 7–8 weeks in the flowering period in each cropping cycle and then harvested. The stock plants, cuttings derived from them, vegetative plants and flowering plants representing different genotypes were tested for HLVd presence at various times during this study, which was conducted during 2021–2023.

### 4.2. Molecular Detection of Hop Latent Viroid (HLVd)

Samples of plant tissues ca. 40–50 mg were used for total RNA extraction. They were ground to a powder with liquid nitrogen and processed using the Qiagen RNeasy Plant Mini Kit (cat. #74904) (Qiagen, Inc., Toronto, ON, Canada) according to the manufacturer’s instructions. The final RNA product was eluted with 52 µL nuclease-free H_2_O. The QIAGEN OneStep RT-PCR Kit (cat. #210212) was used for reverse transcription and PCR amplification. The reaction mixture contained 13 µL of water, 5 µL of 5× reaction buffer, 1 µL of dNTPs (10 mM), 1.5 µL each of HLVd primers [2]: F-5′-atacaactcttgagcgccga-3′, R 5′-ccaccgggtagttTcccaact-3′, 2 µL of RNA template and 1 µL of enzyme mix, resulting in a total volume of 25 µL. All PCR amplifications were performed in a MyCycler thermocycler (Biorad Laboratories, Mississauga, ON, Canada) with the following programme: 30 min at 50 °C, 15 min at 95 °C, followed by 35 cycles of 30 s at 94 °C, 30 s at 58 °C, 60 s at 72 °C and final extension at 72 °C for 10 min. The resulting PCR products were run on a 1% TAE agarose gel and images were captured with E-gel imager (Life Technologies, Carlsbad, CA, USA). Bands of the expected size (ca. 256 and 512 bp) were purified with QIAquick Gel Extraction Kit and sent to Eurofins Genomics (Eurofins MWG Operon LLC 2016, Louisville, KY, USA) for sequencing. The resulting sequences were compared to the corresponding HLVd sequences from the National Centre for Biotechnology Information (NCBI, Bethesda, MD, USA) GenBank database to confirm identity. In each PCR reaction, a positive control (RNA from a confirmed infected plant) and a negative control (water control or RNA from a healthy plant) were included.

### 4.3. Detection of HLVd in Stock Plants and in Flowering Plants

A sampling study was conducted on stock plants represented by 10 cannabis genotypes that were maintained within the dedicated greenhouse nursery area and which ranged in age from 2 to 6 months. The objective was to determine at which sampling position(s) on the plant the presence of the pathogen could be confirmed. Leaves were collected at various positions from the bottom, middle and top of the canopy and root samples were obtained by slicing open the cocofibre bag and removing visible roots using a pair of forceps. All samples were placed inside plastic bags and then transferred to Eppendorf tubes in the laboratory and stored at −80 °C until the molecular analyses for HLVd were conducted, as described above.

In an additional study of flowering plants of one genotype (A5) grown in a research trial, symptoms of HLVd infection were observed within the first 2 weeks of the flowering period. These symptoms included overall stunted growth of the affected plants, as well as reduced growth of the inflorescence stems and reduced overall size of the inflorescence (Figure 1). A total of 20 symptomatic plants were sampled at random by obtaining segments of inflorescence stems and subjecting them to RT-PCR analysis as described above. Similar samples were taken from 5 healthy-appearing neighbouring plants from the same greenhouse compartment.

### 4.4. Effect of Ribonuclease Enzymes on HLVd Integrity

RNA was extracted from leaves of HLVd-infected cannabis stock plants using an extraction kit (RNeasy Mini Kit, Qiagen) and subjected to several enzymatic treatments after which RT-PCR and gel electrophoresis were performed to confirm the presence/absence of bands. The laboratory-grade enzymes tested were RNase A (Qiagen) and RNase R (LGC Biosearch Technologies, Montreal, QC, Canada). One or two units of enzyme were first applied to 50 µL of extracted RNA and incubated at room temperature (23–25 °C) for 10–20 min. Subsequently, the two enzymes were incubated with leaf extracts from infected plants without RNA extraction. One unit was added to 50 µLof sap and incubated at room temperature for 10–20 min. An additional replicate treatment with enzyme was also incubated at 60 °C for the same time periods.

### 4.5. Determining HLVd Structural Configurations Using Mfold

The computational programme mfold [37] was used to visualize secondary structures of the HLVd molecule. The programme algorithm calculates the most thermodynamically stable configuration of an RNA sequence by predicting how suboptimal folding may occur and how bases could pair up to minimize free energy. The programme considers Watson–Crick and wobble base pairing and evaluates possible secondary structures. The complete HLVd RNA sequence (GenBank accession no. OQ420426), retrieved from the NCBI database, was submitted in FASTA format into mfold for structure predictions. To explore alternative structures, eight different initial ΔG free energy values were applied as starting parameters (−95.30, −93.70, −92.40, −92.20, −91.80, −91.40, −91.20, −91.00), allowing mfold to generate a structure at each initial ΔG level. The folding temperature was carried out at 37 °C. The graphical structures were presented in dot–bracket notation and thermodynamic parameters (Supplementary Figure S1). These variations in ΔG provided insights into the range of possible secondary structures that the RNA could adopt.

### 4.6. Transmission of HLVd Through Cuttings from Infected Stock Plants

Stock plants of four genotypes confirmed to be infected with HLVd by RT-PCR as described above (‘S3’, T8, ‘M1’ and ‘B2’) were transferred to an indoor growing room and maintained at a temperature range of 23–27 °C, relative humidity of 50–60% and 24 h supplemental lighting provided by Sunblaster T5HO lamps [32]. After 3 weeks, cuttings were obtained from actively growing shoots of each genotype and rooted in rockwool cubes as described above. The cuttings from each genotype were placed in separate trays. At the time of emergence of the first roots from these cuttings (about 10–14 days later, considered as time = 0 days), duplicate root samples were taken sequentially at 0, 4, 8 and 12 days from each genotype and transferred to Eppendorf tubes and stored at −80 °C until molecular analyses for HLVd were conducted. Samples of roots were collected again at 14 days after root emergence (28 days after cuttings were obtained) and analyzed for the presence of HLVd. A comparison of root development and frequency of emergence was made between cuttings from HLVd-positive and -negative plants.

### 4.7. Transmission of HLVd Through Roots of Infected Plants

#### 4.7.1. Kloner Experiments

For experiments involving HLVd transmission in Kloners, experimental conditions were set up as described by Punja et al. [8]. Briefly, the Kloners contained a recirculating nutrient solution with misting inside a container within which up to 24 cuttings could be rooted and grown. The containers were disinfested with a 2% hydrogen peroxide solution and rinsed. One confirmed HLVd-infected rooted cutting of genotype ‘B2’ and a rooted cutting of the same genotype confirmed to be HLVd-negative by RT-PCR were placed at opposite ends inside the Kloner, ensuring there was no physical contact. The plants were left to grow over a 2-week period during which the root systems remained physically separated from each other while allowing recirculating nutrient solution to flow around them. All Kloners were maintained under indoor growing conditions as described above. Root and leaf tissues were sampled from each plant after 2 weeks of growth and assayed for the presence of the viroid by RT-PCR. The experiment was repeated with a second genotype ‘K4’ under the same conditions.

#### 4.7.2. Greenhouse Experiments

Two groups of vegetative plants of genotypes ‘S3’ and ‘B2’ (four weeks after the initiation of rooting from cuttings) that tested positive or negative for HLVd were placed on 54 cm × 28 cm × 6 cm trays at the extreme ends, providing a 0.5 m distance between them. Twice daily, the trays were flooded with tap water for 5 min and then the excess water was drained off. Two infected plants and three healthy plants of each genotype were placed in each tray, with three replicate trays, and maintained under greenhouse conditions. Samples of roots and leaves from all plants of each genotype were analyzed for HLVd presence after 2 weeks. Water samples from the flooded trays were also tested for the presence of the viroid at the end of the experiment, as described below. The experiment was conducted twice.

### 4.8. Distribution of HLVd in a Trial Greenhouse Environment

To assess the distribution of HLVd at various locations in the greenhouse environment, samples consisting of swabs and nutrient solution were taken. Swabs were made of the surface of visible roots on vegetative plants placed on propagation tables and of the surface of these propagation tables using the sterile Whirl-Pak Hydrated PolyProbe™ sampling bag with sampling sponge pre-moistened with 10 mL of HiCap™ Neutralizing Broth) (BO1592WA) (ColeParmer Canada, Quebec, QC, Canada). Samples of nutrient solution (10 mL) were obtained from pooled solution on tables, drainage solution and solution released from emitters. The swabs were placed inside sterile plastic bags and transported to the laboratory, where the liquid was wrung out by squeezing with a sterile roller into a Petri dish. The various samples of nutrient solution were either used directly or following concentration. To concentrate, the eluate was filtered, 500 μL at a time, through the spin column of a RNeasy Plant Mini Kit (Qiagen) and spinning at 8000 g for 1 min and discarding the flow-through until all 10 mL of eluate was used. All washing steps were followed according to the RNeasy Plant Mini Kit manufacturer’s instructions. The column was then eluted with 30 μL of water followed by re-elution of the same 30 μL with 1 min spins at 8000 g. Positive control samples consisted of crushed roots from HLVd-positive plants. All samples were subjected to RT-qPCR, as described below.

RT-qPCR was performed on a Bio-Rad CFX96 instrument using TaqPath™ 1-Step Multiplex Master Mix (No ROX) (Thermo Fisher Scientific, Waltham, MA, USA). The primers/probe for HLVd detection and cannabis ubiquitin primer/probe used are described elsewhere [2]. The reagents were brought to room temperature prior to use to prevent infrequent non-specific signal increases found when master mixes are prepared on ice. Each 20 μL reaction mixture contained 5 μL of 4 × TaqPath 1-Step Multiplex Master Mix (No ROX; Thermo Fisher Scientific), 1 μL of primer/probes mix and 2 μL of extracted total nucleic acid. The final concentrations of primers and probes were 0.3 μM (target primers), 0.05 μM (target probes), 0.15 μM (internal control primer) and 0.05 μM (internal control probe). The cycling programme on a Bio-Rad CFX96 instrument was as follows: uracil-DNA glycosylase incubation at 25 °C for 2 min; reverse transcription at 53 °C for 10 min; polymerase activation at 95 °C for 2 min; and 40 cycles of PCR at 95 °C for 3 s and 60 °C for 30 s (signal acquisition). The filter combinations were 465–510 (FAM; EF1a P), 540–580 (HEX; HLVd P1) and 610–670 (Cy5; HLVd P2).

### 4.9. Determining the Limits of Detection of HLVd by RT-qPCR

Synthetically derived full-length HLVd controls of known concentration were obtained and the copy number was calculated using the viroid’s molecular weight and Avogadro’s constant. A stock solution of 10^7^ copies of the viroid per μL was prepared and serially diluted 1:10 six times to a final concentration of 10 copies per μL. Next, 100 μL of each serial dilution was mixed with 900 μL of homogenized tissue (100 mg of stem tissue) from an HLVd negative plant. The homogenized tissue was prepared by using a TissueLyser (QIAGEN) at 25 Hz for 1 min in screw-cap tubes with 2.3 mm diameter zirconia-silica beads and 1.0 mm diameter glass beads (BioSpec, Bartlesville, OK, USA). The homogenized tissue was resuspended in 900 μL of extraction buffer (2% CTAB, 2% PVP40,000, 25 mM EDTA pH 8, 100 mM Tris-HCl pH 8, 2.5 mM NaCl) pre-warmed to 65 °C prior to adding to each sample and incubated at 65 °C for 10 min prior to the addition of the synthetic HLVd dilution. The homogenate mixed with the synthetic HLVd serial dilution was centrifuged at max speed (>16,000× *g*) for 5 min at 4 °C and the supernatant was transferred to a new sterile 1.5 mL Eppendorf tube. An equal volume (750 µL) of chloroform isoamyl alcohol (24:1) was added and centrifuged at max speed (>16,000× *g*) for 5 min. The supernatant (550 uL) was transferred to a new 1.5 mL Eppendorf tube and 0.6 volumes of cold isopropanol were added and centrifuged again at max speed (>16,000× *g*) for 5 min. Isopropanol was decanted and about 650 µL of 70% ethanol was added onto the pellets, centrifuged at max speed (>16,000× *g*) for 3 min, and air-dried before re-suspending into 40 µL of nuclease-free water. Twelve replicate PCR reactions were carried out using 1 μL of each serial dilution mixed with HLVd-negative stem tissues and averaged. The limit of detection was determined at the point at which all 12 replicates were detected below a PCR cut-off threshold of 35 cycles. A series of control dilutions was made using synthetic HLVd in nuclease-free water and subjected to the same PCR conditions as the samples that were mixed with plant tissues (Supplementary Figure S2).

### 4.10. Transmission of HLVd by Artificial Inoculation

#### 4.10.1. Inoculum Preparation

Several studies were conducted to demonstrate the transmission of HLVd from infected plants to viroid-free plants. To ensure a continual source of viroid inoculum, infected stock plants of several genotypes (‘W12’, ‘D7’, ‘X12’) were maintained in an indoor growing room in 10 L pots containing a cocofibre–perlite mix (3:1) for periods of up to 16 months. These plants received hydroponic nutrient solution as needed and were pruned regularly to prevent excessive growth. In addition, deep water culture of infected stock plants in 12 L plastic buckets containing hydroponic nutrient solution aerated with air stones and an aquarium pump was also established. To prepare inoculum, leaf or root samples (2 g) from these infected stock plants were crushed in a mortar and pestle with 10 mL of sterile distilled water to produce plant extracts (infectious sap) that were used for inoculation. All plants to be used for inoculation were first tested by RT-PCR to confirm they were viroid-free. These plants were kept physically separated from the infected stock plants in a different location of the growing room.

#### 4.10.2. Cut-Stem Inoculations

Vegetative cuttings of five genotypes (‘B2’, ‘K4’, ‘C9’, ‘W12’ and ‘M1’) were rooted and grown hydroponically in Kloners, as described previously. After 4 weeks, a stem segment was removed from a leading stem and the exposed stem surface was dabbed with a Q-tip that was dipped in the infectious plant extract (approx. 200 uL was applied). The plants were grown in the Kloner for an additional 6 weeks after inoculation under a 24 h photoperiod. To detect HLVd, samples of leaves and roots were obtained at 4 and 6 weeks after inoculation from all five genotypes and analyzed by RT-PCR. There were 4 replicate plants of each genotype. The experiment was conducted twice.

In further inoculation experiments, rooted plants of genotypes ‘B2’ and ‘S3’ were grown in pots containing cocofibre–perlite for 6 weeks. A stem segment was removed from a leading stem on each plant as previously described and the exposed surface was dabbed with a Q-tip dipped in infectious plant extract. The plants were maintained in the pots in the indoor growing room for an additional 6 weeks and fertilized as needed. At 2 and 5 weeks after inoculation, leaf and petiole samples were taken from various positions around the plant, in addition to root samples, and tested for the presence of HLVd to establish the distribution of the viroid. The plants were left to grow for 10 weeks to observe for symptom development.

To determine whether HLVd was transmitted through the physical abrasions made to leaves, plants of ‘S3’ were damaged by rubbing leaves between two fingers to result in visible tearing or a pair of scissors was used to trim the ends of some leaves to expose cut edges. Plants receiving both types of wounding were inoculated on the cut surfaces with infectious sap and left to grow for 4 weeks. Leaf samples were taken from positions distal to the site of inoculation representing newly developed leaves and assayed for the presence of HLVd. Control plants were wounded but not inoculated.

### 4.11. Effect of a 12:12 H Photoperiod on Viroid Spread

Rooted cuttings of genotype ‘H6’ confirmed to be HLVd-positive by RT-PCR were grown in cocofibre–perlite for 4 weeks under a 24 h photoperiod in the indoor growing environment. A set of 2 infected plants was then transferred to a Conviron growth chamber set at 24 °C and a photoperiod of 12 h light–12 h dark while a second set was placed under continuous 24 h lighting. The plants were grown for an additional 3 weeks and each set was examined for symptom development and overall morphology. In a subsequent experiment, 4-week-old plants each of genotypes ‘M1’, ‘K4’ and ‘S3’ that were demonstrated to be HLVd-negative by RT-PCR were wound-inoculated on cut stems as described previously, and after 24 h, a set of 2 plants was subjected to a 12:12 h photoperiod or a 24 h photoperiod. Leaf samples from the bottom, middle and top of the plant, as well as root and flower samples (only for the 12:12 h plant), were obtained after 21 days and tested for the presence of HLVd by RT-PCR. In addition, samples were analyzed for viroid levels in these tissues by RT-qPCR, as described previously, at 12 and 21 days after transfer to 12:12 h and compared to those at constant 24 h light. The experiment was repeated using an autoflower genotype, which automatically initiates flowering regardless of photoperiod, to determine if the duration of light in this genotype had an impact on the spread of the viroid. Samples of leaves from the bottom, middle and top of the plant, as well as root and flower samples, were obtained after 21 days and tested for the presence of HLVd by RT-PCR.

### 4.12. Evaluation of Additional Hosts for Susceptibility to HLVd

To assess the ability of HLVd to infect Solanaceous host species, seeds of tobacco (*Nicotiana tabacum* ‘Samsun’) and tomato (*Solanum lycopersicum* ‘Celebrity’) were germinated in planting mix containing cocofibre–perlite under a 24 h photoperiod. Once the seedlings had reached a height of 10 cm, they were transferred to 8.5 cm^2^ pots and grown for an additional 2–4 weeks in the indoor room prior to inoculation. Inoculations were performed by removing a leaf or stem segment with a sterile scalpel and inoculating the exposed cut surface at the site of excision with infectious leaf extract, as described previously. Plants were grown for periods of 6–11 weeks as needed until HLVd was detected. Samples of roots, lower leaves, middle leaves and top leaves were taken from inoculated and uninoculated control plants for HLVd detection using RT-PCR, as described previously. Sampling times varied according to plant species but began at 2 weeks after inoculation (for roots) and continued until 11 weeks (for roots and leaves). Five plants each of tobacco and tomato were included in the experiment, which was repeated once.

In an additional series of experiments involving tomato plants ‘Celebrity‘, 4-week old plants grown in 10 cm diameter pots were placed in a tray and inoculated with a mixture of infectious plant extract and nutrient solution (1 mL sap–100 mL solution) which was poured into each pot. This was repeated 4 subsequent times (each time adding 15 mL/pot) at 3 day intervals to ensure the roots came into contact with the inoculum. Plants were allowed to grow for an additional 4–6 weeks and tested for HLVd presence. Control plants received nutrient solution without sap.

### 4.13. Assessing Flower Tissues and Trichomes for HLVd Presence

Plants of genotypes ‘C9’ and ‘D7’ growing in the flowering room of a cannabis greenhouse and displaying symptoms of stunted growth and reduced inflorescence development characteristic of HLVd infection [16] were tested for the presence of HLVd in various tissues. Entire inflorescences from symptomatic plants were subjected to drying conditions to reduce the moisture content to 10–12% [4,17]. Inflorescences from HLVd-negative plants were similarly harvested and dried. To obtain a trichome preparation, samples of HLVd-positive and HLVd-negative dried inflorescences were gently crushed by hand and the material was sifted through a set of 5 stacked sieves (1.18 mm, 600 um, 250 um, 150 um and 75 um sizes). The dried material retained on the 150 um sieve consisted of trichome stalks and glandular heads, while that retained on the 75 um sieve consisted primarily of smaller trichome heads. To confirm the composition of these materials, subsamples were examined under the magnification of a dissecting microscope (50×). RT-PCR analyses were conducted on duplicate samples obtained from each of the 150 um and 75 um screenings.

### 4.14. Detection of HLVd in Extracted Resinous Material

Dried inflorescences from HLVd-infected plants of genotypes ‘H6’ and ‘H10’ were subjected to several different processes commonly used to extract total cannabinoids and terpenes to yield resinous oils. These processes included (i) CO_2_ extraction to produce “crude oil”, (ii) CO_2_ extraction plus ethanol extraction followed by roto-evaporation to yield “winterized oil” and (iii) winterized oil with terpenes added back to produce “full spectrum oil”. All resinous samples extracted by each of these processes were used for HLVd detection. Prior to extraction of RNA, the thick resin was suspended in a lysis buffer (Buffer RLC, Qiagen) and incubated for at 60 °C for 30 min. Extraction of RNA was then performed using an extraction kit (RNeasy Mini Kit, Qiagen) and subjected to RT-PCR and gel electrophoresis.

### 4.15. Transmission of HLVd Through Seeds and Pollen

#### 4.15.1. Hemp

Hemp seeds of a dual-purpose fibre/grain genotype from an experimental breeding trial were planted in a cocofibre–perlite medium in the indoor environment with 24 h lighting. Prior to planting, a random sample was tested for presence of HLVd by RT-PCR. To demonstrate transmission of HLVd from inoculated seeds to seedlings, artificially inoculated hemp seeds that were soaked in infectious sap for 10 min were used. Emerging hemp seedlings from non-inoculated and inoculated seeds were sampled at the cotyledonary and first true leaf stages after 10–14 days and tested for HLVd presence. Additional testing was also conducted on young true leaves sampled from 3–5 week old plants. Following continued growth, some plants produced inflorescences which contained male flowers after 6 weeks. Samples of flowers, anthers and pollen were then collected from these hemp plants in an Eppendorf tube for HLVd detection. In addition, pollen samples were examined under the scanning electron microscope (SEM) following previously published procedures [17].

#### 4.15.2. Cannabis

To demonstrate HLVd transmission through cannabis seeds, 4-week-old female plants of genotype ‘G11’ originating from HLVd-positive cuttings were experimentally sprayed with a 3 mM solution of laboratory-grade silver thiosulphate thrice at 7-day intervals to induce male flower formation [75]. After 3–4 weeks, clusters of male flowers appeared at the internodes of treated plants and produced pollen that was collected and used to fertilize female plants of cannabis genotype ‘M1’ by dusting pollen manually onto the stigmas. HLVd presence in the ‘G11’ plants was confirmed from root, leaf, whole-flower and anther samples taken from two silver-treated plants. Samples of anthers and pollen were also examined by SEM. Seeds derived from the ‘M1’ × ‘G11’ cross were collected at maturity and a random sample of 16 seeds was tested for HLVd presence using RT-PCR. A separate batch of seeds was then germinated on moist paper towels and the emerging roots, primary root, and cotyledonary tissues were sampled at various times during the 5–12 day-period following radicle emergence and tested for HLVd. Additional testing was also conducted on true leaves of seedlings that developed from seeds planted in potting medium after 3 weeks.

### 4.16. Survival of Hop Latent Viroid in Plant Tissues and on Surfaces of Utensils

#### 4.16.1. Plant Tissues

To determine the extent of the survival of HLVd (estimated by the integrity of the RNA molecule to undergo RT-PCR amplification) in cannabis tissues, leaves and roots from the HLVd-positive stock plants grown as a source of inoculum were used. Leaf tissues were crushed in a mortar and pestle and extracts of sap were left to dry in weighing boats placed on tables in the indoor growing room for periods of 0.5, 1, 3 and 5 h, or for 1, 3, 5 and 7 days. In addition, crushed leaf extract was rubbed onto PPV gloves and left to dry for the same periods of time. Intact leaves and roots were also left to dry for periods of 1, 2, 3 and 4 weeks at room temperature. After each experiment was completed, 40–50 mg of tissues were subjected to RT-PCR to determine if HLVd was detectable.

#### 4.16.2. Utensils

Commonly used utensils in the laboratory or indoor growing room were tested for the presence of HLVd. These included disposable plastic pestles used to grind plant tissues for RNA extraction (Bel-Art, SP Scienceware, Wayne, NJ, USA), plastic containers used to store hydroponic nutrient solution, and hand-held plastic watering cans. The plastic pestles were re-used multiple times, each time undergoing an autoclave cycle of 15 min at 121 °C with 0.9 KPa of pressure before re-use. The pestles were left to soak in water for 4 h and the water was tested for HLVd. The buckets were used for periods of up to 4 months for the storage of nutrient solution, which was prepared fresh every week. The watering can was used to deliver nutrient solution manually, frequently coming into contact with plants, and was dipped into the nutrient reservoir each time it was used. The surfaces of both the bucket and watering can were swabbed and tested for the presence of HLVd. Samples of nutrient solution were also analyzed for the presence of HLVd. They were used directly for RNA extraction and subsequent RT-PCR, as described previously.

### 4.17. Effect of Temperature and UV-C Treatment on HLVd Survival

Tissues from HLVd-positive stock plants were used to assess the effect of heat and UV-C treatments on HLVd survival. Leaves were removed at random from these plants while roots were harvested from plants grown in deep water culture and a slurry of 0.5 g of roots in 5 mL of water was prepared. For temperature treatments, small pieces of leaves and the root slurry (750 uL) were placed inside 2 mL centrifuge tubes. All tubes were then immersed in a temperature-controlled water bath at temperatures ranging from 30 to 80 °C for 15 or 30 min. An additional set of samples was subjected to 90 and 95 °C for periods of 1–5 min by placing the tubes on a heat block. For UV-C treatments, leaves and root segments were placed in 60 × 15 mm plastic Petri dishes and then irradiated for 0, 2 or 5 min using a hand-held lamp (Cleanlight^TM^ Pro. Honselersdijk, The Netherlands) that delivered approximately 3–6 mJ/cm^2^ of radiation [13]. After each treatment, the tissue samples were transferred to Eppendorf tubes and stored at −80 °C until used for HLVd detection by RT-PCR. Control tissues were not subjected to any treatment and left at room temperature for comparable periods of time and then frozen until used.

### 4.18. Effect of Disinfectants and Chemicals on HLVd Integrity

#### 4.18.1. Whole Root Tissues

Root tissues were obtained from plants grown in deep water culture. Four sanitizing products were tested for their effect on HLVd stability. The commercial greenhouse products tested were Virkon S (potassium peroxymonosulfate + sodium chloride, LanXESS, Toronto, ON, Canada) (tested at 0.25, 1.0, 1.5 and 2.0%) and Zerotol (27% hydrogen peroxide, BioSafe Systems, East Hartford, CT, USA) (tested at 0.33, 0.5, 1.0 and 2%). The laboratory sanitation products tested included household bleach (Chlorox, containing 8.25% sodium hypochlorite NaOCl) (tested at 5, 10, 15 and 20%) and hypochlorous acid (Ecologic Solutions, Brooklyn, NY, USA) (tested at 0.1, 0.25, 0.5, 1.0 and 2.0 ppm). The chemicals were made up to varying concentrations in water and added to 60 × 15 mm Petri dishes containing 0.1 g of root tissue and left for a 2 min exposure, after which the solutions were poured out and the treated tissues were immediately transferred to Eppendorf tubes and frozen at −80 °C until used.

#### 4.18.2. Crushed Leaf Extracts

Infected leaves were crushed as described previously and the extract was used to soak 90 mm diameter filter papers (Whatman #5). The discs were dried in a laminar flow hood for 2 h, after which 5 mm diameter discs were punched out and transferred to 60 × 15 mm Petri dishes, with 5 discs per dish. Each dish received one of the four chemical products previously used on root tissues. A range of concentrations was tested with a 1 min exposure as follows: Virkon at 2–4%, Zerotol at 2%, bleach at 2.5–20% and hypochlorous acid at 200–1000 ppm. Additional treatments of 20% skim milk powder (Carnation brand) for 20 min, 70% ethanol for 10 min, UV-C for 30 to 90 s, RNase A for 10 or 20 min, with and without incubation at 60 °C, and a filtered extract from a *Bacillus subtilis* culture for 10 min, were also evaluated. After each treatment, the discs were transferred to Eppendorf tubes and immediately frozen at −80 °C until used. Positive and negative controls were included. A series of treatments were also applied to infectious sap containing *C. sativa* mitovirus (CasaMV1) [2] to determine which treatments could degrade the RNA of the virus.

#### 4.18.3. Contaminated Water

Leaves (2 gm) from an HLVd-infected plant were crushed in a mortar and pestle with 10 mL of water and 2.5 mL of the sap extract was added to 500 mL of water in a beaker. The mixture was subjected to the following chemical (1 min) or UV treatments (1–5 min) after which a 500 uL sample of the mixture was transferred to an Eppendorf tube and placed at −80 °C for subsequent RT-PCR analysis for viroid integrity. The treatments included bleach (8.25% NaOCl) at a final concentration of 5, 10 and 20% (*v*/*v*), Virkon at a concentration of 2% (*w*/*v*), Zerotol at a concentration of 2% (*v*/*v*), hypochlorous acid at a concentration of 600 ppm, or exposure to UV-C from a CleanLight^TM^ Pro hand-held lamp for 1, 2 or 5 min.

### 4.19. Effect of Meristem Tip Culture

Infected plants representing 8 cannabis genotypes were included in this study. They were confirmed to be HLVd-positive by RT-qPCR as described previously [16]. Cuttings were obtained from these plants and taken back to the laboratory, where shoot tips measuring 1 cm in height were excised. They were surface-sterilized by immersion for 10 min in a 10% (*v*/*v*) solution of commercial bleach (containing 5.25% NaOCl) with 0.05% (*v*/*v*) Tween 20 and rinsed three times in sterile water. Meristems measuring 0.2–0.4 mm in length with two or fewer leaf primordia were excised under a dissecting microscope. They were transferred onto tissue culture medium as described in Shi et al. [38], adopted from Lata et al. [76] containing full-strength DKW salts (PhytoTech Labs, Inc. Lenexa, KS, USA), B5 vitamins (PhytoTech Labs, KS, USA), 3% sucrose (Thermo Fisher Scientific, Inc. Waltham, MA, USA), 0.7% (*w*/*v*) agar and 2 μmol·L^−1^ meta-topolin (PhytoTech Labs.) The medium was adjusted to pH 5.7 and autoclaved at 121 °C for 20 min. The containers with explants were incubated at 22 ± 2 °C under an 18 h photoperiod with 50 μmol·m^−2^·s^−1^ of full spectrum light and sub-cultured at four-week intervals to fresh medium. In total, up to 8–10 surviving meristems were transferred from each genotype. The developing shoots were tested for the presence of HLVd every six weeks for a period of 6 months, as described in [2].

### 4.20. Summary of Hop Latent Viroid Spread

Based on the results from this study, the proposed avenues by which HLVd can spread in a hydroponic cannabis greenhouse facility are shown in Figure 19. Infected stock plants produce infected cuttings, from which HLVd can spread through roots to give rise to infected flowering plants and ultimately spread through pollen and seed (Figure 19).

## Figures and Tables

**Table 1:** Detection of Hop latent viroid on the surfaces of swabbed plant roots, on propagation tables and in recirculated water samples in a cannabis-growing facility using RT-qPCR.

Sample Source	RT-qPCR Value (Ct)	HLVd Present (+)/Absent (−) ^a^
Crushed roots from symptomatic plants		
Sample A	24.44	+
Sample B	25.69	+
Swabbed roots of symptomatic plants		
Sample A	32.55	+
Sample B	34.77	-
Swabs of table surface under symptomatic plants		
Sample A	25.08	+
Sample B	26.91	+
Nutrient solution samples draining from tables		
Sample A	32.00	+
Sample B	33.33	-
Nutrient solution samples from grooves on table		
Sample A	29.81	+
Sample B	29.72	+
Nutrient solution emitted from nozzles		
Sample A	31.89	+
Sample B	31.06	+

^a^ Presence/absence of HLVd was based on a Ct cut-off value of 33 or lower.

**Table 2:** Effect of two different photoperiods on the distribution and levels of Hop latent viroid in various tissues of three cannabis genotypes, as determined by RT-qPCR.

Genotype	Tissue Source	Days Post-Inoculation	Photoperiod	
			24 h Light RT-qPCR Value	12:12 h Light–Dark RT-qPCR Value
‘M1’	Roots	12	28.5	24.62
	Mid-leaf	12	0	31.25
	Top Leaf	12	26.0	19.71
	Flower	21	n/a	17.71
‘K4’	Roots	12	30.25	26.60
	Mid-leaf	12	0	0
	Top leaf	12	0	24.70
	Flower	21	n/a	19.72
‘S3’	Roots	12	25.6	21.3
	Mid-leaf	12	31.1	29.4
	Top leaf	12	22.5	18.7
	Flower	21	n/a	16.2

**Table 3:** Incidence of Hop latent viroid in tissue-cultured cannabis plants derived from meristems originating from eight genotypes of *C. sativa* infected with HLVd.

Infected Plant	Avg. Ct of HLVd—Positive Cuttings	No. of Meristems	Frequency of HLVd–Negative Plants (%)	Avg. Ct of HLVd–Positive Plants
Genotype A	20.56 ± 0.76	10	4 (40%)	15.12 ± 0.60
Genotype B	22.60 ± 1.42	10	7 (70%)	14.33 ± 0.40
Genotype C	15.83 ± 0.30	12	1 (8.33%)	17.05 ± 3.45
Genotype D	25.18 ± 1.04	10	10 (100%)	-
Genotype E	22.55 ± 0.45	12	3 (25%)	15.15 ± 1.94
Genotype F	17.07 ± 0.15	12	4 (33.33%)	16.97 ± 0.22
Genotype G	15.18 ± 0.27	11	0 (0%)	16.16 ± 0.78
Genotype H	23.67 ± 0.64	12	8 (66.66%)	17.71 ± 1.64
Total		91	37 (40.66%)	

Meristems about 0.4 mm in size were aseptically harvested from shoot tips taken from cuttings obtained from plants that tested positive for HLVd. The meristems were cultured as described by Shi et al. [38] in individual tissue culture vessels and tested every six weeks for HLVd presence using RT-qPCR as described by Punja et al. [2]. The data represent results 6 months after the experiment was initiated.

## Data Availability

All data generated or analyzed during this study are included in this published article.

## Supplementary Materials

The following supporting information can be downloaded at: https://www.mdpi.com/article/10.3390/plants14050830/s1, Figure S1: Secondary structure of hop latent viroid; Figure S2: Relationship of Ct values in RT-qPCR to copy numbers of hop latent viroid.
