# Supplementary material for: Transmission, Spread, Longevity and Management of Hop Latent Viroid, a Widespread and Destructive Pathogen Affecting Cannabis (Cannabis sativa L.) Plants in North America
Source: Plants (Basel). 2025 Mar 6;14(5):830. doi: 10.3390/plants14050830 (PMC11902214; doi:10.3390/plants14050830)
Supplement: Supplementary file 1 [file plants-14-00830-s001.zip › plants-3437884-supplementary.pdf]

Figure S1. Secondary structure of hop latent viroid. **(a)** The hop latent viroid (HLVd) secondary structure predicted from the full 256 bp sequence using RNA-fold (GenBank accession OQ420426). The proposed structures show the complementary base-pairing and the stem loop structures, as well as the secondary structure of the RNA molecule based on different ΔG. Structure at an initial ΔG of -93.70 kcal/mol (left). Structure at an initial ΔG of -92.20 kcal/mol (right). **(b)** Predicted RNA configurations of HLVd RNA (GenBank accession OQ420426) generated using mfold at an initial ΔG of -91.00 kcal/mol. Magnified view of complementary base-pairings and stem loop structures of the configuration predicted at an initial ΔG of -91.00 kcal/mol is shown. Red and blue connections indicate Watson-Crick base pairings (A-U and G-C) and green connections indicate wobble base pairings (G-U).

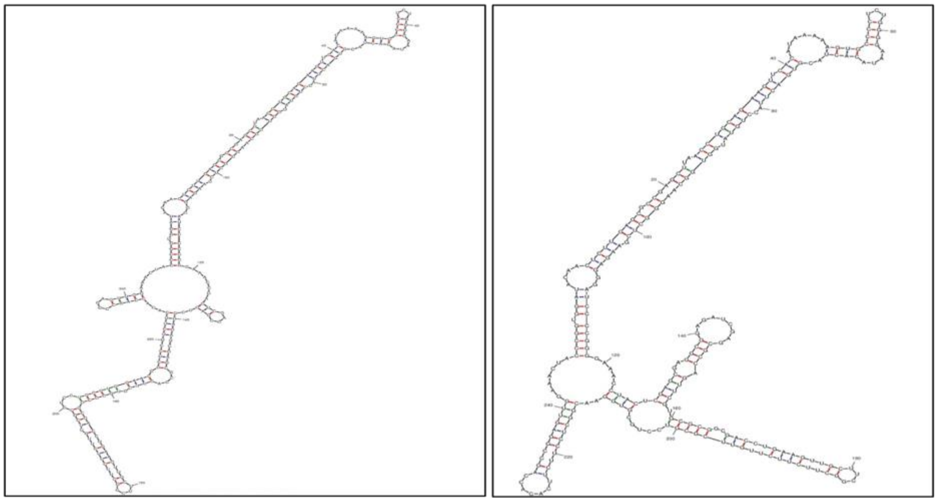

(a)

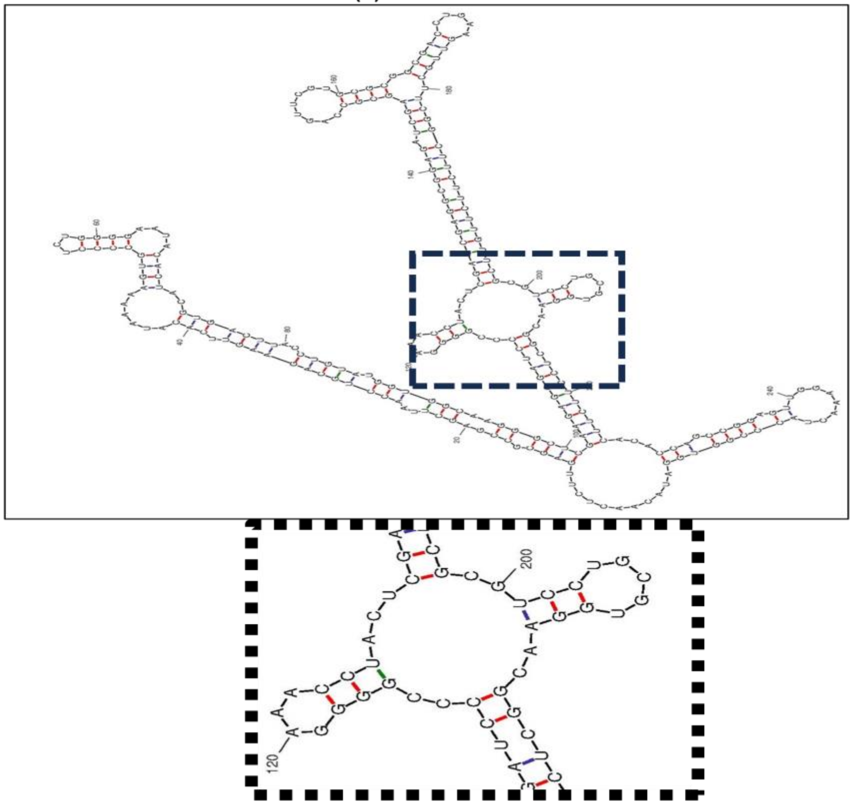

(b)

Figure S2. Relationship of Ct values in RT-qPCR to copy numbers of hop latent viroid. **(a)** Detection in plant sap. The figure shows the relationship between Ct values in RT-qPCR reactions and the approximate number of HLVd viroid copies present in plant tissues. Synthetically derived full-length HLVd controls of known concentration were used, and the copy number was calculated using the viroid's molecular weight and Avogadro's constant. Twelve replicate PCR reactions were carried out using 1 µL of each serial dilution. The limit of detection was determined at the point at which all 12 replicates were detected below a PCR cut-off threshold of 35 cycles. **(b)** Standard curve in water. A stock solution of 10^7 copies of the viroid per µL was prepared and serially diluted 1:10 with water six times to a final concentration of one copy per µL. Five replicate PCR reactions were carried out using 1 µL of each serial dilution. The standard curve was generated for each run using the primers in a multiplex assay. The cycle threshold (Ct) was calculated as the intersection between an amplification curve and the baseline of the background signal. The Ct values were plotted against the Log viroid copy number to generate the standard curve for each HLVd target gene and linear regression was performed.

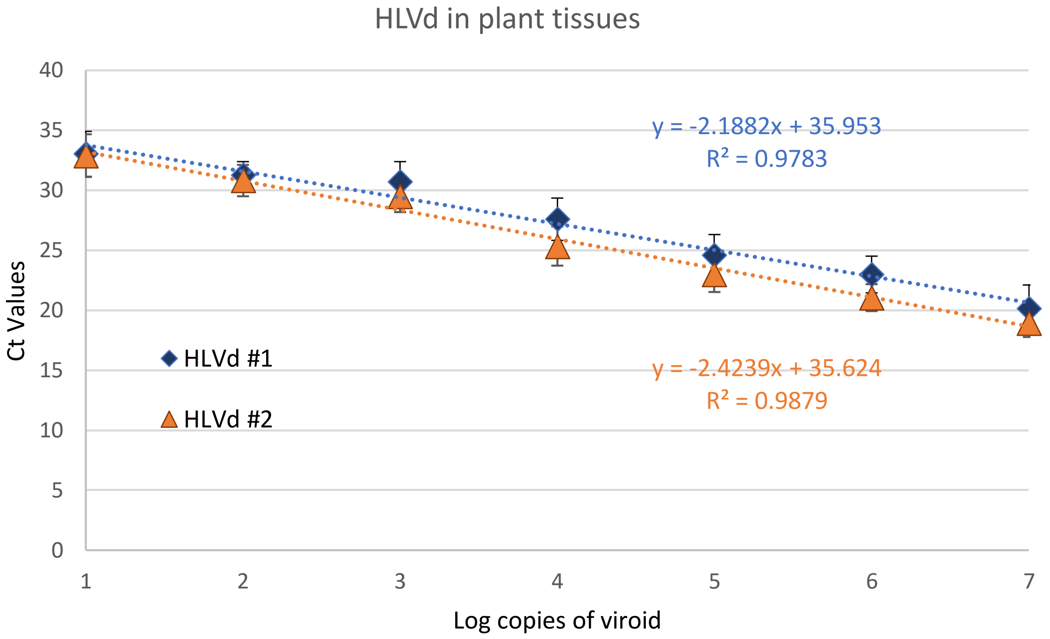

(a)

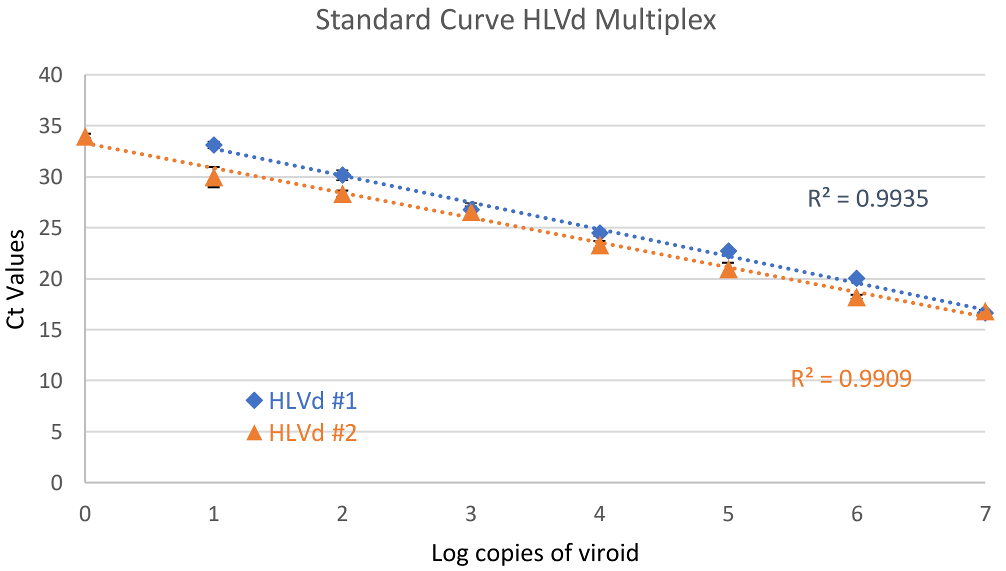

(b)
